# Inflammatory cells and their non-coding RNAs as targets for treating myocardial infarction

**DOI:** 10.1007/s00395-018-0712-z

**Published:** 2018-12-06

**Authors:** Mira Jung, Michael Dodsworth, Thomas Thum

**Affiliations:** 1 Institute of Molecular and Translational Therapeutic Strategies (IMTTS), Hannover Medical School, Carl-Neuberg-Str. 1, 30625 Hannover, Germany; 2 National Heart and Lung Institute, Imperial College London, London, UK

**Keywords:** Immune response, Myocardial infarction, Neutrophil, Macrophage, Lymphocyte, Therapeutic strategy, Inflammation

## Abstract

Myocardial infarction triggers infiltration of several types of immune cells that coordinate both innate and adaptive immune responses. These play a dual role in post-infarction cardiac remodeling by initiating and resolving inflammatory processes, which needs to occur in a timely and well-orchestrated way to ensure a reestablishment of normalized cardiac functions. Thus, therapeutic modulation of immune responses might have benefits for infarct patients. While such strategies have shown great potential in treating cancer, applications in the post-infarction context have been disappointing. One challenge has been the complexity and plasticity of immune cells and their functions in cardiac regulation and healing. The types appear in patterns that are temporally and spatially distinct, while influencing each other and the surrounding tissue. A comprehensive understanding of the immune cell repertoire and their regulatory functions following infarction is sorely needed. Processes of cardiac remodeling trigger additional genetic changes that may also play critical roles in the aftermath of cardiovascular disease. Some of these changes involve non-coding RNAs that play crucial roles in the regulation of immune cells and may, therefore, be of therapeutic interest. This review summarizes what is currently known about the functions of immune cells and non-coding RNAs during post-infarction wound healing. We address some of the challenges that remain and describe novel therapeutic approaches under development that are based on regulating immune responses through non-coding RNAs in the aftermath of the disease.

## Introduction

Cardiovascular diseases (CVDs) remain the leading cause of death world-wide, accounting for 31% of all fatalities in 2016 (WHO, June 2016). An important contributing factor is our incomplete understanding of the processes by which tissue is remodeled after a myocardial infarction (MI). One hallmark of the disease is the recruitment of a diverse range of immune cells which are recruited into the infarcted heart and modulate both innate and adaptive immune responses [111]. During the initial phase after MI, for example, inflammation plays a causal role in remodeling the left ventricle (LV) and is accompanied by a rearrangement of myocytes, extracellular components and vessels [67].

Infiltrating cells exhibit specific patterns of spatiotemporal distribution and activity [212] while carrying out an active, sequential crosstalk with each other and other cardiac cells. This creates a highly complex regulatory landscape [132, 142] that plays an important role in proper post-MI cardiac healing [149]. Inflammatory processes may also cause hypertrophy, fibrosis and other types of cardiac damage which can subsequently lead to heart failure [102]. Optimum recovery, thus, requires a timely and selective modulation of inflammation [48, 49], but the heterogeneity and functional diversity of immune cells pose challenges in attempts to target inflammation as a therapeutic strategy. Successful approaches will require a more comprehensive understanding of the spatiotemporal coordination of immune responses in post-MI tissue.

Here, we highlight the temporal dynamics of immune cells during post-MI LV wound healing and consider the therapeutic potential of engineering such cells. We focus particularly on recent findings regarding the roles of noncoding RNAs (ncRNAs) in regulating immune cell functions. An additional focus is the growing list of molecules known to participate in the recruitment, activation and polarization of immune cells after MI, opening new avenues for pharmaceutical manipulation that may lead to improved forms of immunotherapy for MI patients.

## Inflammatory responses post-MI and ncRNAs derived from immune cells

In the aftermath of MI, monocytes, lymphocytes and other immune cells sequentially orchestrate wound healing procedures that are crucial in preserving cardiac functions [48]. In the infarcted myocardium, the death of massive numbers of cardiomyocytes leads to damage-associated molecular patterns (DAMPs) or inflammasomes [47]. These serve as danger signals that are recognized by the Toll-Like Receptor (TLR) or Nod-Like Receptor (NLR) families, which recruit immune cells to the region of the infarct and activate an inflammatory response [89, 144, 176, 181]. There has been increasing evidence that heart failure alters the expression of specific ncRNAs, suggesting that they play a role in the development and aftermath of cardiovascular diseases [122, 211]. ncRNAs comprise diverse RNA molecules that are not translated into proteins. The two predominant types are microRNAs (miRNA) (generally defined with a size of 22–23 nts) and long non-coding RNAs (lncRNA) (> 200 nts). A study has described an association between non-coding RNAs and post-MI disease status; interestingly, the strongest associations found in this study involved non-coding RNAs implicated in processes related to inflammation and immunity, suggesting that they play important roles in post-MI immune responses [107].

### Neutrophils

Neutrophils (polymorphonuclear granulocytes; PMNs) are leukocytes that play a crucial role in innate immunity by eliminating foreign pathogens through degranulation, oxidative mechanism, and other mechanisms. An accumulation of neutrophils is characteristic of the acute inflammatory response [93], and an increase in the number of circulating leukocytes has been considered a hallmark of systemic inflammation in MI patients [137]. Leukocyte numbers are strongly associated with high mortality rate in patients following MI [124], suggesting that these cells are linked to adverse cardiac remodeling. Neutrophils are recruited into the infarct myocardium as CD11b^+^Ly6G^+^F4/80 − for mice and CD11b^+^CD16^+^CD62L^+^ for humans [147, 207] (Table 1).

**Table 1:** The expression phenotype and their function of Immune cell subsets during cardiac remodeling post-MI

	Human subset	Mouse subset	Function	Strain	References
Neutrophil	CD11b^+^, CD16^+^, CD66b^+^HLA-DR	CD11b^+^, Ly6G^+^, F4/80^−^	Digest pathogens	C57BL/6	[147, 207]
Monocyte					
Mo1	CD14 high, CD16^−^CCR2^+^	Ly6C^high^	Homing, initiation of inflammatory process	C57BL6, apoE^−/−^	[133, 155, 159]
Mo2	CD14 high, CD16^+^CCR2^+^		Phagocytosis, vascular repair.		
Mo3	CD14 low, CD16^+^CCR2^−^	Ly6C^low^	Anti-inflammatory response, tissue repair, angiogenesis		
Macrophage					
M1	Ly6^hi^CD206^−^CD204^−^	MHCII^hi^CD11c^hi^CCR2^hi^	Pro-inflammatory. ECM digestion	C57BL/6, Trib1^−/−^	[51, 162]
M2	Ly6c^low^, CD206^+^CD204^+^	CD206^+^, F4/80^+^, CD11b^+^	Anti-inflammatory, fibrosis		
Dendritic cell	Plasmacytoid CD68, CD303^+^	CD11c^+^CD11b^−^	Ag presenting cell, induce T-cell immunity	C57BL/6	[228]
	Myeloid DCs CD1C^+^				
	Myeloid DCs CD141^+^				
Lymphocyte					
Th1	CD3^+^, CD4^+^, IFN-γ^+^	CD3^+^, CD4^+^, IFN-γ^+^	Differentiation of infiltrated monocytes	C57BL/6, CBAIJ, BALB/c	[228]
Th2	CD3^+^, CD4^+^, IL-4^+^	CD3^+^, CD4^+^, IL-4^+^	Inducing B-cell antibody isotype, anti-inflammatory response	BALB/c ByJ, B10.D2 mice	[43]
Th17	CD3^+^, CD4^+^, IL-17A^+^	CD3^+^, CD4^+^, IL-17A^+^	Pro-inflammatory response	C57BL/6, BALB/c, DO11.10 TCR-transgenic mice	[168]
Treg	CD3^+^, CD4^+^, CD25, CD127	CD3^+^, CD4^+^, CD25, Foxp3	Suppressor T cell, balancing immune response	C57BL/6	[160, 228]
B cells	CD3^−^, CD19^+^	CD3^−^, CD19^+^	Mediate humoral immune response by producing antibodies	C57BL/6J, CD45.1	[212, 230]

*LncRNA (lnc-)* long non coding RNA, *miRNA (mir-)* microRNA, *PBMC* peripheral blood mononuclear cells, *DC* dendritic cells

**Fig. 1:**
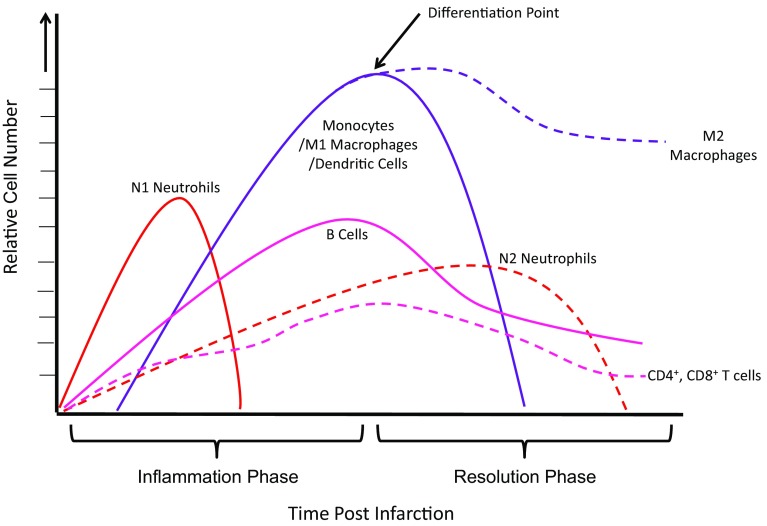
Temporal dynamic of immune cells during post-MI healing

PMNs are the first immune cells to infiltrate the infarcted myocardium after MI [229]. They migrate into the infarct within hours after permanent coronary occlusion in mice, reaching a peak at days 1–3 and dropping to normal level at days 5–7 post-MI [117, 118] (Fig. 1). After infiltration, PMNs are activated through the expression of recognition receptors such as TLRs or NLRs. Once active, PMNs can digest pathogens through several mechanisms which subsequently initiate inflammatory responses. These include the secretion of antimicrobial granule contents such as reactive oxygen species (ROS) or matrix-degrading proteinases, or by forming neutrophil extracellular traps (NETs), in addition to other microbicidal mechanisms that are capable of mediating tissue injury [5, 118, 142, 229]. An increased neutrophil–lymphocyte ratio (*N/L* ratio) has been identified as a marker for adverse outcomes in patients suffering from ST-segment elevation post myocardial infarctions (STEMI) [90, 137]. Recent findings from Nalbant et al. offer insights into this ratio and adverse cardiac remodeling post-MI: MI patients exhibit elevated neutrophil counts compared to healthy counterparts, while these groups display no differences in lymphocyte counts [134]. These findings suggest that neutrophil infiltration might be a promising therapeutic target for better outcome post-MI. Neutrophils also play an important role in the recruitment and activation of monocytes/macrophages at later post-MI time points, suggesting that their role in wound healing goes beyond directly killing pathogens [50].

### Neutrophil derived ncRNAs

Recent studies have shown that ncRNAs produced by neutrophils have regulatory effects on their functions during inflammatory responses [82, 204]. An example is miR-223, the most abundant miRNA in neutrophils, which is critical for their differentiation from precursor cells [83, 204]. The expression of this microRNA has not been studied specifically in neutrophils that infiltrate cardiac tissue, though high levels of its expression are highly correlated with the development of heart failure [199]. In heart samples from both human patients who have experienced heart failure and a hypertrophic mouse heart model [achieved through the use of transverse aortic constriction (TAC)], this miRNA is massively up-regulated compared to healthy controls [199]. The systemic over-expression of miR-223 in mice has a negative impact on several pathogenic parameters in vivo, including the expression of genes linked to cardiac stress, heart size and levels of interstitial fibrosis [199]. The fact that miR-223 is known to have inflammatory effects [175] suggests that these disease phenotypes are at least partially influenced by a dysregulation of inflammatory processes.

miR-5192-5p, which is linked to atherogenesis, is expressed at significantly higher levels in circulating neutrophils from patients with MI compared to those derived from a healthy group [198]. Neutrophils also highly express miR-15b, which has been shown to exhibit anti-apoptotic effects on cells during cardiac remodeling after MI [74, 112, 209]. Like other cellular systems that regulate gene expression, miRNAs can play either beneficial or detrimental roles in processes of health and disease, depending on the molecule involved and its range of targets in a specific developmental or pathological context. While a function for miR-15b in the context of a cardiac-specific inflammation has not yet been described, it has been shown to regulate a system inflammatory response following Japanese Encephalitis infections, which is strongly suggestive of a direct link [222]. Other noncoding RNAs that are abundant in neutrophils and have been implicated in cellular dysfunction include miR-491-3p, miR-34b, miR-595, miR-328, miR-1281 and miR-483-3p, all of which exhibit alterations in expression in the senescent state [204].

In addition to the intrinsic effects of miRNAs on the neutrophils that produce them, they can be transferred through micro-vesicles to endothelial cells in a process that affects atherogenesis. miR-150 and -223 have been shown to undergo this type of transfer [58]. This suggests a novel potential strategy for treatment based on targeting micro-vesicles as ncRNAS are delivered from cell to cell.

lncRNAs produced specifically by granulocytes, such as HOXA cluster antisense RNA 2 (HOXA-AS2) and Morrbid, are also of interest due to their association with neutrophil survival through the regulation of apoptosis [180]. These molecules prolong the lifespan of neutrophils by regulating TNF-related apoptosis (HOXA-AS2) and the transcription of the pro-apoptotic gene Bcl2l11 (Morrbid) [96]. As neutrophils’ survival is strongly associated with prolonged inflammation following MI, cardiac neutrophil-specific expression of these lncRNAs could be of interest. Jiang et al. have shown that the expression of lncRNAs produced by neutrophils can be influenced by disease: in a comparison between groups of patients suffering from arthritis and those experiencing clinical remission on medication (CRM), they found a differential expression of 38 lncRNAs [82]. Although the functions of these lncRNAs are largely not known, this suggests that gene regulation by lncRNAs may mediate and fine-tune neutrophilic transcriptional action for specific inflammatory responses, potentially including MI.

### Monocytes and macrophages

Abnormal levels of blood mononuclear cells (PBMCs) including monocytes, macrophages and lymphocytes have been associated with adverse inflammatory responses following MI [46]. Of these, monocytes are the most abundant, and not only serve as a source of myeloid for the differentiation of macrophages and dendritic cells, but also present antigens, regulate other immune cells, and serve additional functions [80]. They make up 10% of the total human and 4% of mouse blood leukocytes and are major players in the innate immune system [42]. Mice have two subsets (inflammatory Ly6C^high^ and anti-inflammatory Ly6C^low^ phenotypes) exist [133], while humans possess three subtypes of monocytes, classified by the relative expression of CD14, CD16 and CCR2: Mon1 (classical; CD14^high^CD16^−^CCR2^+^), Mon2 (inter-mediate; CD14^high^CD16^+^CCR2^+^) and Mon3 (non-classical; CD14^low^CD16^+^CCR2^−^) [133] (Table 1). Classical human and Mon1 mouse monocytes induce pro-inflammatory responses and exhibit high phagocytosis, whereas non-classical/Mon3 monocytes express anti-inflammatory mediators [155, 159]. In the steady state, the inflammatory circulating forms account for 50–60% of the total circulating monocytes in mice (Ly6C^hi^CCR2^high^CX3CR1^low^CD62 L^+^) and 80–90% in humans (CD14^high^CD16^−^) [116]. MI stimulates adrenergic signaling that triggers the bone marrow and spleen to produce new monocytes that are recruited into the heart [42]. Within 24 h post-MI, the weight of the spleen decreases by 50% accompanied by a depletion of monocyte numbers as a result of their departure from the spleen’s monocyte reservoir [116, 171]. Chemokine receptors induce the recruitment of monocytes into the infarct region; inflammatory Ly6c^hi^ monocytes are recruited early on post-MI in a CCR2-dependent manner, whereas anti-inflammatory Ly6c^low^ monocyte recruitment is dependent on Cx3cr1 and occurs later. These monocytes differentiate into macrophages or dendritic cells (DCs) in response to various chemokines and growth factors released from the injured tissue [172, 212].

**Fig. 2:**
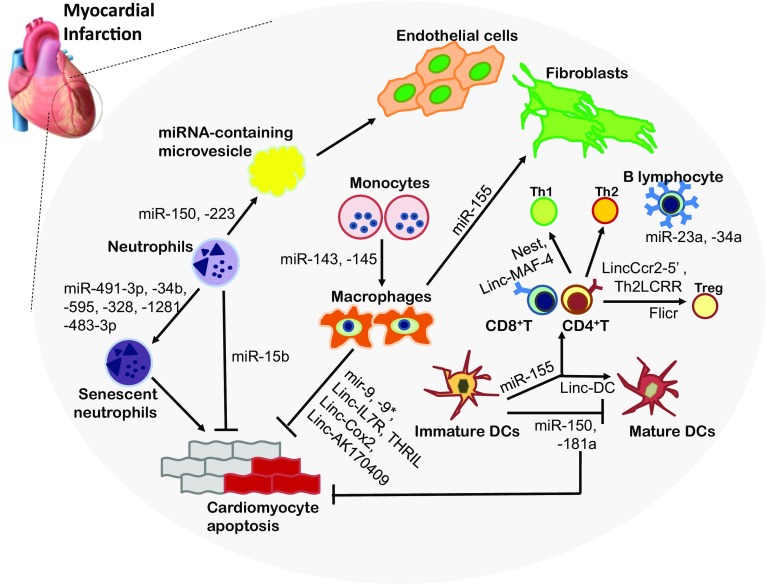
Immune cell-derived ncRNAs. *miRNA (mir-)* microRNA, *Linc (linc-)* long intergenic noncoding RNAs, *DC* dendritic cells, *Treg* regulatory T cells

Macrophages are our body’s primary phagocyte, indiscriminately destroying a huge variety of pathogens and also clearing debris from apoptotic cells. They also trigger an immune cascade through both antigen presentation and the release of signaling molecules and enzymes [59]. Macrophages play crucial roles during LV remodeling post-MI due to their involvement in every stage of the wound healing process, including inflammation, resolution, and maturation phases [63, 79, 99]. Infiltration begins at day 1 and peaks at 3–5 days post-MI (Fig. 1) [212]. Four subsets of cardiac macrophages are distinguished in mice through the presence of surface markers: Ly6C, MHCII, CD11c and CCR2 [44]. To date, most studies have oversimplified the character of macrophages into either pro-inflammatory M1 or anti-inflammatory M2 phenotypes. Mouse MHCII^hi^CD11c^hi^CCR2^hi^ cells are described as M1 and CD206^+^F4/80^+^CD11c^+^ as M2 [162]. In humans, Ly6^hi^CD206^−^CD204^−^ are described as M1 and Ly6c^low^CD206^+^CD204^+^ macrophages as M2 phenotypes [51]. Immediately after MI, there is a predomination of M1 inflammatory macrophages which secret pro-inflammatory cytokines and MMP (matrix metalloproteinases), but over time this phenotype gradually switches to that of M2 macrophages which promote repair. Later during the maturation phase, these macrophages regulate the activation of fibroblast and endothelial cells [111]. This plasticity of macrophages has attracted many researchers to strategies involving immune reprogramming; therapeutic interventions, however, would need to carefully consider factors such as the time point post MI and the desired M1/M2 ratios. These issues may contribute to our poor understanding of the clinical outcome of such approaches (Fig. 2).

### ncRNAs derived from monocytes or Macrophages

Paahuleva et al. reported differences in the expression of miRNAs in circulating monocytes between patients with MI and healthy controls. MI patients exhibited an upregulation of miR-143 and -145, involved in macrophage differentiation and activation, from peripheral blood monocytes [220, 221]. Interestingly, miR-143 has been shown to have anti-inflammatory effects during allergic rhinitis by inhibiting IL-13 secretion, suggesting a similar role in cardiac monocytes or macrophages [177]. Monocyte-derived miRNAs also influence neighboring cells, as the expression of miR-126 in circulating monocytes is highly linked to angiogenesis and vascular inflammation [76]. Furthermore, miR-126 circulating in plasma has been proposed as a diagnostic marker for myocardial infarction [114]. An additional miRNA, miR-155, was elevated in cardiac macrophages in injured hearts, and its upregulation had a direct impact on fibroblast proliferation in post-MI remodeling [197]. The depletion of miR-155 in macrophages is correlated with decreased levels of CCL2, a chemokine that recruits monocytes, suggesting that macrophage-derived miR-155 expression might serve as a therapeutic target which could suppress both fibrosis and inflammatory responses [135]. Monocyte activation leads to an upregulation of miR-9, miR-9*, lnc-IL7R and THRIL, which suppress several genes that serve as regulators of the inflammatory response in immunity [12, 30]. Of further interest is NF-κB, a transcription factor that plays one of the most important roles in activating inflammatory genes. Enhanced NF-kB signaling in macrophages is associated with excessive inflammations and higher rates of mortality post-MI. A recent study from Covarrubias et al. used CRISPR/Cas-based screening to identify macrophage-derived lncRNAs Cox2 and AK170409 as NF-kB regulators. Knocking down these lncRNAs result in a significant reduction of proinflammatory gene expression [26]. Reprograming macrophages with these ncRNAs could provide a novel therapeutic approach toward fine-tuning the transcription of numerous inflammatory genes following infarction.

### Dendritic cells

DCs represent the other major cell type derived from monocyte differentiation. They phagocytose pathogenic material and migrate to the lymph nodes, where they present antigens to CD4^+^ T-helper cells, a key step in establishing immunological memory [10]. There is increasing evidence for a role for DCs during post-MI wound healing. Significantly reduced numbers of circulating DC precursors have been observed in patients with MI, presumably due to a correlated increase in DC recruitment into the infarct myocardium [97, 214]. In humans, higher numbers of DCs were observed in the myocardium of a patient who died due to MI compared to that of a patient who died by car accident, suggesting that DC infiltration correlates with post-MI mortality rates [97].

DCs originate from the bone marrow, but unlike macrophages, they do not have a dedicated phagocytic character, and their lifespan is short [10]. An accumulation of CD11c^+^ CD11b^+^ DC in the infarct was shown to peak at 7 days after ligation in both mouse and rat models with permanent occlusion [10, 228] (Fig. 1). A tripartite subdivision of the human DC lineage cells in blood has been proposed: one plasmacytoid group (CD68 CD303^+^) and two myeloid group types (CD1C^+^ and CD141^+^) [228] (Table 1). As efficient antigen-presenting cells (APC), DCs play an important role in linking innate and adaptive immune responses, and thus activate T-cell-induced immunity [37]. Recent studies have observed an association of DCs with increased numbers of macrophages and lymphocytes including T cells [97]. This indicates that DCs play a crucial role in the recruitment and activation of immune cells in the myocardium residing. Here, their numbers must be carefully balanced to mount an effective immune response without causing excessive further damage to cardiac tissue following MI. DCs affect macrophage polarization and lymphocyte differentiation, two functions that should be considered when contemplating approaches that target them therapeutically, particularly given their potential impact on the immune microenvironment [69, 109].

### ncRNAs derived from dendritic cells

miR-155 is one of the most abundant and crucial miRNAs in the hematopoietic system. It can regulate the expression of other DC miRNAs such as miR-455 and is involved in the activation of the cells [40]. In a manner similar to macrophages, the depletion of DC-derived miR-155 inhibits inflammatory responses because cells fail to produce CCL2. DCs highly express a number of lncRNAs that play important roles in DC differentiation and DC-mediated T cell activation [200]. Upon exposure to endotoxins, DCs release exosomes enriched in miRNAs into the spleen and initiate an inflammatory response [205]. Few studies have been devoted to the effects of DC-derived ncRNAs on post-MI remodeling.

### Lymphocytes

The lymphocyte family is comprised of T cells, B cells and natural killer cells which arise from a common lymphoid progenitor. Unlike immune cells derived from the myeloid lineage, T cells and B cells have a major influence on adaptive immunity, tailoring a unique immune response through cell-mediated and humoral (i.e., antibody-related) mechanisms [210]. T cells can again be further categorized into CD4^+^ helper T cells, CD8^+^ cytotoxic T cells, and regulatory T cells (Treg). In contrast to observations for neutrophils and monocytes, the absence of lymphocytes is associated with worse outcome in MI patients [137]. Decreased CD4^+^ count strongly contributes to a drop in lymphocytes following MI.

A significant decrease in the CD4^+^–CD8^+^ ratio can be shown 24 h after MI and can be used to predict poor outcomes in patient with MI [13]. The depletion of CD4^+^ T cells results is characterized by increased inflammation and fibrosis, suggesting they play a role in repairing wounds post-MI [70]. B lymphocytes interact with monocytes after MI and accelerate their infiltration and migration into the infarct, which injures the tissue [230]. Natural killer (NK) cells are another pivotal player in innate immunity that have been shown to be involved in post-MI healing. NK cells are generally downregulated following infarction; their sustained presence results in reduced inflammation, however, suggesting a protective role in wound healing post-MI [7, 84].

### ncRNAs derived from lymphocytes

Many miRNAs are clustered with other miRNAs which are produced from a single primary transcript; interestingly, one third of human miRNAs are clustered. The most well-known miR-cluster, miR-17–92 cluster (miR-17, -18, -19, -20, -92) is expressed at high levels during B- and T cell development and promotes the survival of lymphocytes and has been associated with autoimmune pathologies [210]. It has also been implicated in a wide range of cardiovascular pathologies including myocardial infarction, where miR-19 and other components are thought to stimulate cardiomyocyte proliferation following the injury [60]. MiR-155, an abundant hematopoietic miRNA, is associated with T-cell differentiation. It has beneficial effects on these cells, in contrast to its effects on macrophages and DCs, which indicates that the customization of miRNAs as therapeutic targets will require taking into account cell-specific aspects of their functions. A deficiency in the production of miR-155 impairs the development of Th17 and Treg cells, indicating that miR-155 is crucial for CD4^+^-mediated immune suppression alongside its roles in neutrophils and monocytes/macrophages [14, 92]. Higher level of miR-155 in aged miR-146 KO mice has been associated with an accumulation of activated T cells [73]. In the same study, Ruozhen Hu et al. created mice with T-cell-specific miR-155 deficiencies (Cd4-cre Mir155fl/fl) to determine its role in these immune cells. They discovered that the expression of the miRNA was specifically required for the proper development of T-cell lineages in the spleen and lymph nodes [73]. miR-155-driven immune regulation might have a protective effect on the injured myocardium following tissue death by diversifying its immune cell repertoire [110]. Another cluster named miR-23a, which includes miR-27a, and miR-24, is highly expressed in B cells alongside miR-34a to regulate their differentiation and maturation [94, 151]. miR-23a’s pro-hypertrophic role involves NAFTc3 signaling in cardiomyocytes and has been linked to cardiac pathologies [108].

lncRNAs that appear to be specifically regulated in lymphocytes include Nest (Nettoie Salmonella pas Theilers’s) and Linc-MAF-4. Both are expressed in T cells and NK cells, and are important for Th1 lineage differentiation [192]. lincCcr2-5′ and Th2LCRR promote Th2 polarization [6, 68], whereas a Treg-specific lncRNA called Flicr disturbs cellular activity and promotes autoimmunity by regulating the key transcription factor FoxP3 [216]. It is unclear whether the activities of these ncRNAs change in lymphocytes following cardiac injury, but the question is cleary worth pursuing.

## Strategies for therapeutic interventions that target immune cells

### Inhibiting inflammatory responses

#### Suppressing the recruitment of immune cells

##### Recruitment of neutrophils

Recent studies have established a positive correlation between the infiltration of neutrophils into damaged cardiac tissue and the severity of phenotypes seen in patients with MI [137, 227]. MI triggers an infiltration of these immune cells into the tissue [193]. Their recruitment is triggered by elevated levels of the chemokines macrophage inflammatory protein-2α (MIP-2α) and leukotriene B4 (LTB4) [91]. Increased numbers of neutrophils and their secretory molecules are strongly associated with adverse cardiac remodeling and the size of the infarct post-MI, suggesting that the modulation of their infiltration might be a promising strategy to regulate inflammation [138, 157].

Limitations of neutrophil infiltration can be achieved through the administration of metoprolol, a β1-adrenergic-receptor (ADRB1) antagonist, which is widely used in treating ischemic reperfusion (IR) damage. This effectively reduces the size of the infarct and protects against cardiac dysfunction [4, 136]. A recent study from García-Prieto et al. demonstrated that treatment with metoprolol did not change infarct size in a neutrophil-depleted model, indicating that the post-MI effects of the drug are dependent on these cells [53]. Treatment with the JAK3 inhibitor JANEX-1 [4-(40-hydroxyphenyl)-amino-6,7-dimethoxyquiazoline], attenuated the migration and infiltration of neutrophils, and resulted in a decreased infarct size with improved post-MI cardiac function [139]. A specific inhibitor of Nicotinamide phosphoribosyltransferase (Nampt), FK866, has also been shown to impair CXCL2-induced neutrophil recruitment and neutrophil-mediated inflammatory responses, reducing infarct size after MI [127].

##### Macrophage recruitment/macrophage migration

Macrophages are major effector cell types in the inflammatory response after ischemic insults. Several studies have established a direct link between high numbers of macrophages in the infarct and adverse LV remodeling post-MI [86, 92, 187]. This has led to methods to visualize inflammatory status along with cardiac remodeling by assessing macrophage states based on their stimulators or the molecules they secrete [32]. Chemokine receptors including CCR2, CXCR6, and the macrophage migration inhibitory factor (MIF) contribute to the recruitment of macrophages into the ischemic myocardium, and levels of these molecules have been associated with adverse ventricular remodeling and cardiac dysfunction following infarction [19, 52, 87]. Accordingly, strategies to inhibit macrophage infiltration into the infarct myocardium have revealed successful results in an in vivo model after MI. CCR2 knock out mice showed significant decreases in inflammation along with better remodeling post-MI [87]. The deletion of MIF also results in reduced infarct size and cardiomyocyte apoptosis [19, 52]. Splenic monocytes also contribute to the infiltration of macrophages into the myocardium post-MI. Increased levels of circulating inflammatory monocytes in the blood and macrophages in the heart have been associated with a prolonged inflammatory response and adverse LV remodeling in Hmox1^−/−^ mice. Conversely, splenectomized Hmox1^−/−^ mice exhibited improvements in cardiac function post-MI. This suggests that the modification of splenic monocytes might serve as a means of blocking the recruitment of macrophages [182]. These results suggest that interfering with macrophage recruitment through the regulation of chemokines or other means might be of interest as a therapeutic strategy for MI. This would entail risks, however, since a depletion of macrophages unexpectedly leads to cardiac dysfunction post-MI because they apparently play a crucial role during cardiac remodeling.

##### Dendritic cell recruitment

Atherosclerosis, MI, and other cardiovascular diseases are accompanied by an increase in the infiltration of DCs into the injured myocardium; their influence appears to be exclusively beneficial [212]. Numbers of these cells are significantly reduced in the infarct area in patients with cardiac rupture post-MI, suggesting that DCs might help to inhibit ruptures [130]. These cells also balance proportions of M1 and M2 macrophages in the regulation of inflammatory responses. Low DC counts in patients with rupture are accompanied by a heightened pro-inflammatory phenotype and impaired fibrosis [130]. As APCs, DCs also play a crucial role in linking the innate and adaptive immune response [166]. Increased numbers of DCs correlate with MHC II expression and T-cell contacts post-MI, suggesting that they help to regulate adaptive immune responses during wound healing post-MI [97]. Deeper insights into these diverse roles of DCs might be useful in promoting post-MI wound healing.

Despite these findings, clinical trials based on inhibiting inflammatory cell infiltration in MI patient have so far failed to improve cardiac functions or reduce the size of infarcts. There are several possible explanations: (1) animal models might not sufficiently mimic patient physiology, (2) these methods might fail to prevent the assembly of the terminal complement complex in STEMI patients undergoing Primary Percutaneous Coronary Intervention (PPCI), or (3) interactions with other organs or other complex aspects of the disease are not present in the studies [141]. These findings suggest that targeting the inflammatory response requires more than simply inhibiting the recruitment of specific types of immune cells.

#### Regulating the maturation of immune cells

##### Dendritic cell maturation

The maturation of DCs occurs in a step-wise manner involving (1) the uptake of an antigen (Ag), (2) Ag presentation (APC), (3) migration and (4) interactions with T cells. Steady-state blood contains numerous immature DCs, called circulating dendritic cell precursors (DCPs) [10]. Immature DCs can take up antigens through phagocytosis or endocytosis, but they lack the ability to activate T cells. During pathological processes, pathogen- or damage-associated molecules (PAMPs or DAMPs) trigger an expression of chemokine receptors such as CCR1, CCR2 and CXCR1 on the surfaces of DCs. Interactions with their partner chemokines stimulate the TLR signaling pathway, which causes DCs to mature from Ag-capturing cells to APCs which are capable of interacting with T cells [10]. This maturation is accompanied by changes in morphology and functions and cells’ phagocytic capacity disappears [10, 170]. Under hypoxic conditions, mature DCs stimulate the inflammatory response and affect cardiomyocyte apoptosis [225].

Mature DCs can migrate into lymphoid tissue, where they become resident cells and present Ags to T cells [103]. CCR7 and CXCR4 play key roles in homing DCs to the regional lymph node [36, 150, 156]. There mature DCs activate T cells through orchestrated signals, including increased Ag presenting molecules (MHC class I or II), costimulatory molecules (CD40, CD86) and adhesion molecules (CD11a,b, CD50, CD54) [10, 33, 170]. Mature DCs act as master regulators of T-cell auto-reactivity in the heart. Quantities of Th1 and Th17 cells increase in the presence of conventional DC (cDC1) post-MI, while Treg cells accumulate in the presence of a different dendritic subtype (cDC2) in the steady state [186]. cFos is an important regulator of DC maturation, which in turn regulates the activation of T cells. A c-Fos treatment group exhibited reduced infarct size, which might be due to a resaturation of DC maturation [41, 218]. Recent work from Liu et al. has documented a process by which exosomes released from mature DCs induce a proliferation of T helper cells (CD4^+^ T cells) in the infarct at days 5–7 post-MI, leading to improved cardiac function [109]. The regulation of mature DCs and their secretome is crucial for a balanced immune response, making them popular targets for therapy.

### Resolution of inflammation

#### Clearance of apoptotic cells

##### Neutrophil apoptosis

The influence of neutrophils in MI has received little attention due to their low numbers and short life spans in ex vivo conditions (5–10 h for human and 4 h for mouse PMNs) [164]. A recent study by Pillay’s group, however, has demonstrated a prolonged lifespan for PMNs under in vivo conditions (5.4 days for human and 8–10 h for mouse neutrophils) [146]. The differences in these two experimental conditions suggest that inhibitory signals may prevent neutrophil apoptosis in vivo conditions. Following the clearance of pathogens, neutrophils undergo cell death through either necrosis or apoptosis. Neutrophilic apoptosis is a constitutive process that plays an important role in resolving inflammations [16]. Well-known proinflammatory cytokines such as tumor necrosis factor (TNF)-α and interleukin (IL)-1β inhibit apoptosis, leading to prolonged survival of PMNs in MI [28]. Prolonged PMN lifespans led to adverse healing in a patient with chronic heart failure (CHF), involving an increase in endothelial damage [183]. Accordingly, inhibiting MMP-12 diminishes neutrophilic apoptosis, causing a similar progression with exaggerated inflammations in mice after infarction. This supports a strong association between neutrophilic apoptosis and the effective post-MI wound healing [78].

Dying PMNs release “eat me” signals that lead to their engulfment by macrophages, initiating repair and healing processes post-MI [16]. Apoptotic PMNs secrete molecules such as lactoferrin and annexin A1 that further inhibit PMN infiltration and induce macrophage recruitment. The secretion of lactoferrin by PMNs suppresses the release of NET and the recruitment of immune cells into the inflammatory sites, which mitigate secondary damage [105, 140]. Elevated levels of lactoferrin have been considered as a strong predictor of higher risk for fatal ischemic heart disease in patients with diabetes [191]. It is still unclear whether an increase in lactoferrin release acts as a compensatory signal in balancing pro- and anti-inflammatory responses. Another molecule secreted by PMNs, Annexin A1, can regulate further inflammatory events by inhibiting the infiltration of leukocytes and activating apoptosis in neutrophils. This factor simultaneously promotes the clearance of apoptotic cells by macrophages [169].

The degradation of apoptotic neutrophils by macrophages induces an anti-inflammatory response in which macrophages polarize to the reparative phenotype (M2) and the resolution of inflammation accelerates [165]. While there are some benefits to a decrease in neutrophil infiltration, their depletion led to an unexpected worsening of cardiac function and an increase in fibrosis. Inflammation was exaggerated due to significantly decreased levels of phagocytotic markers [16, 71]. This indicates the importance of the engulfment of apoptotic neutrophils by macrophages, particularly in resolving post-MI inflammation. At the same time, this complicates attempts to target the pathogenic functions of neutrophils, because of their beneficial roles in healing infarcts. A potential solution might be the administration of neutrophil gelatinase-associated lipocalin (NGAL), a component of the apoptotic neutrophil secretome which can restore macrophage phagocytosis in neutrophil-depleted mice post-MI [120]. This suggests that the regulation of inflammation by managing or mimicking neutrophil apoptosis might be a useful strategy in the treatment of MI patients.

##### Macrophage phagocytosis

Apoptotic and necrotic cells and debris are engulfed through phagocytosis, as a major function of macrophages during LV remodeling post-MI and a key mechanism for resolving inflammation. Mouton et al. analyzed macrophage physiology over the duration of MI, determining that macrophages isolated from mice 3 days post-MI exhibited an enriched phagocytic and proliferative phenotype [129]. A depletion of MertK, a specific marker of phagocytosis, correlates with prolonged inflammations due to an insufficient clearance of apoptotic cells [196]. MMP-9, the most efficient metalloproteinase during MI insults, decreases macrophage phagocytosis through the degradation of the cell surface antigen CD36. Neutrophils in the infarct have prolonged survival, causing a sustained inflammation that increases LV dilation [35]. Thus, macrophage phagocytosis is required to resolve inflammation.

Recent studies have shown that the M2 phenotype of macrophages is strongly correlated with the cells’ phagocytic ability. Mice in which M2 macrophages have been systematically depleted experience impairments in the clearance of inflammatory cells in the infarcted myocardium, associated with poorer LV remodeling after MI, as well as a reduced ejection fraction (EF) and increase in the size of the infarction [104]. The uptake of dead cells or debris by macrophages induces a release of anti-inflammatory cytokines such as IL-10 and TGF-β, which are M2 macrophage polarization markers. This is in contrast to a decrease in the expression of inflammatory markers such as TNF-α and IL-1β, suggesting that apoptosis contributes to resolving the inflammatory response. Overall, enhanced phagocytosis results in improved cardiac function with decreased LV dilation post-MI [65]. The modulation of macrophage phagocytosis could, thus, be a promising therapeutic avenue for patients with MI [65].

#### Inflammatory cells polarization

##### Neutrophil polarization

PMNs contribute to the reparative phase and homeostasis at later post-MI time points. Unexpected results have emerged from experiments in which neutrophils have been depleted: in mice at day 7 post-MI, this led to an increase in LV dimensions with reduced ejection fraction (EF) and massive fibrosis [71].

Ma et al. were the first to report a possible polarization of neutrophils during post-MI LV remodeling. Similar to macrophages, neutrophils exhibit one of the two distinct phenotypes. The N1 phenotype associates with a pro-inflammatory response and predominates at an early time point, then polarizes over time towards the N2 phenotype, associated with a post-MI anti-inflammatory response [117].

After permanent occlusion in mice, neutrophils express pro-inflammatory cytokines (Tnf-α, Il-1β, Il-12a) or matrix proteinases (Mmp9, Mmp12). These features are characteristic of N1 neutrophils and described as Ly6G^+^CD206^−^. During post-MI wound healing, N1 phenotypes are induced in neutrophils by the presence of DAMPs and initiate inflammation, which leads to post-MI wall thinning. At 5–7 days post-MI, neutrophils express an increased number of anti-inflammatory cytokines, suggesting a switch in their phenotype during post-MI healing. N2 neutrophils are activated as Ly6G^+^CD206^+^ and their numbers gradually increase, peaking at day 7 post-MI. N2 neutrophils are locally activated, hinting at signals that cause a switch between the N1 and N2 phenotypes. This plasticity of neutrophils can be exploited to develop new strategies to modulate inflammation [117], but achieving this will require a more profound study of the exact role N2 neutrophils play in LV remodeling post-MI.

##### Modifying the microenvironment/macrophage polarization

Macrophages are versatile cells which present diverse phenotypes and functions depending on their microenvironments [22]. Following MI, cardiac macrophages in the infarcted area exhibit time-dependent changes in the state of their polarization, associated with both an early pro-inflammatory phase (M1 phenotype) and late reparative phase (M2 phenotype), respectively [45, 55, 129, 212]. A timely resolution of inflammation is crucial for optimal wound healing, which suggests that manipulating the M2/M1 ratio might be a strategy to prevent further damage after an infarct. Modifying the microenvironment has been a strategy used to exploit this plasticity and trigger a switch of phenotypes from M1 to M2. In recent work from our group, the administration of IL-10 triggered an increased polarization of M2 macrophages and led to better LV remodeling, improved cardiac function and stable fibrosis post-MI [86].

Macrophage activation can also be modulated by specific transcription factors with beneficial effects on post-MI LV remodeling. The progression of heart failure can be attenuated through a suppression of interferon regulatory factor 5 (IRF5), a crucial transcription factor in cardiac macrophages, which is downregulated in M1 cardiac macrophages [25]. Wnt signals, which are involved in the differentiation of macrophages, are also induced in the cells by cardiac stress or injury. Inhibiting these signals polarizes macrophages to the M2 anti-inflammatory phenotype, leading to improved cardiac repair [145].

Above, we described the contributions of splenic monocytes on macrophage infiltration into the infarct. Splenic leukocytes, and to a lesser extent macrophages, also play a role in resolving inflammation by producing and delivering specialized proresolving mediators (SPMs) into the infarcted LV [63]. SPMs are generally derived from lipid mediators, and changes in lipid signaling can alter immune kinetics in a way that leaves inflammations unresolved. Lipid metabolic enzymes such as COX and LOX have been shown to play a role in resolving inflammation and its relevant cardioprotective effect on post-MI cardiac healing [88]. The glycoprotein Semaphorin3A (Sema3A), which is secreted by circulating monocytes, can also influence macrophage polarization. Expression of Sema3A significantly increases during later time points following MI, and this is correlated with an up-regulation in the expression of Cx3CR1, a marker for reparative monocytes. Sema3A heterozygotes exhibit poor post-MI progression, which strongly suggests the importance of immune resolution for proper post-MI wound healing [153]. Regulatory T cells (Tregs) are also involved in regulating monocyte/macrophage differentiation. Treatment with anti-CD28 monoclonal antibody (CD28-SA) activates Tregs and promotes the differentiation of macrophages into the M2 phenotype, with positive effects on post-MI LV remodeling [206].

A growing body of evidence suggests that macrophage activation is accompanied by a metabolic shift [100]. While LPS-induced M1 macrophages rely on glycolysis for ATP production, IL-4-induced M2 macrophages incorporate oxidative phosphorylation for the efficient production of energy [27, 184]. A recent study by Van den Bossche et al. suggests that regulating mitochondrial functions could provide novel approaches to reprogramming macrophage polarity. Inhibiting iNOS improved mitochondrial function and prompted the repolarization of macrophages from M1 to M2, potentially an exciting approach toward regulating the cells’ phenotype and post-MI activity [185].

Modifications in the microenvironment of macrophages also contribute to fibrosis and tissue repair. Macrophages isolated from day 7 post-MI infarcts exhibit an increased expression of collagen I and periostin, indicating that macrophages mediate a re-organization of the extracellular matrix (ECM), which is a major characteristic of fibrosis and tissue repair [129]. After an ischemic injury, macrophages produce various growth factors including transforming growth factor β1 (TGF-β1), insulin-like growth factor 1 (IGF-1) and vascular endothelial growth factor (VEGF), which activate the recruitment and proliferation of fibroblasts. During the proliferative phase of the wound healing process, fibroblasts differentiate into myofibroblasts, restoring the lost ECM [208]. A study from Shiraishi’s group showed that M2 macrophages are crucial for post-MI, fibroblast-mediated repair, supporting a scenario in which fibroblast activation preferentially occurs through one macrophage phenotype [162]. This suggests that the modulation of macrophage polarization could simultaneously target processes of both inflammation and fibrosis, two of the major factors in the progression of heart failure after MI.

### Balancing homeostasis

#### Neutrophil extracellular traps (NETs) and neutrophil granule components

Neutrophil extracellular traps (NETs) are networks consisting of DNA or extracellular fibers released from de-granulated neutrophils that induce defense mechanisms against injury [194]. NETosis (i.e., the activation and release of NETs) is a recently described immune action of neutrophils that coordinates wound healing after injury [29]. The high expression of a NET marker (cell-free deoxyribonucleic acid) has been detected following MI, and this strongly correlates with infarct size [3, 31]. Ge et al. demonstrated that NETs could be degraded by treatment with DNase, resulting in an improvement of post-MI cardiac function and pointing to NETs as another novel potential target in treating MI patients [54]. Domingo-Gonzalez’s group showed for the first time that prostaglandin E2 (PGE2) serves as an inhibitory signal for NETosis [38]. In a different study, treatment with PGE2 increased the ability of stem cells to regenerate cardiomyocytes, suggesting that PGE2 plays a positive role in post-MI cardiac repair [72]. If this protection is conferred through an inhibition of NETosis inhibition, NETs would appear to be an attractive target with the aim of improving cardiac repair.

During NETosis, neutrophils can de-granulate, which releases antimicrobial cytotoxins or proteinases that can regulate the inflammatory response. PMN granules are classified into four main types: (1) azurophilic; (myeloperoxidase (MPO), serine proteases, azurocidin, lysozyme), (2) specific; [lactoferrin, neutrophil gelatinase-associated lipocalin (NGAL, lipocalin-2)], (3) gelatinase; (matrix metalloproteinases), and (4) secretory; (complement receptor 1, CD13 CD14, CD16) [71]. MPO activity peaks with the accumulation of neutrophils and inflammatory monocytes, and is correlated with an adverse inflammatory response, which has led to the assessment of MPO activity as a means of imaging post-MI cardiac inflammation [32, 125, 131]. The effects of these proteins on post-MI healing are still being clarified. Some evidence suggests that the neutrophil granule proteins lactoferrin and pentraxin 3 (PTX3) improve cardiac function and infarct size after MI [23, 105, 140, 161]. Contrary to this finding, high levels of MPO in the plasma are correlated with increased mortality after MI, which means that inhibiting the post-MI accumulation of MPO accumulation could improve LV remodeling [2, 125].

The seemingly contradictory results seen for NETs and their granule proteins might be explained by a ‘double-edged sword’ theory by which they not only kill pathogens to the body’s benefit, but also detrimentally sustain the digestion of tissues. A sustained exposure to granule proteins and their oxidation products is ultimately cytotoxic and leads to long-term adverse post-MI LV remodeling [189]. The conclusion is that NETs and secreted proteins are neither exclusively pro- nor anti-inflammatory mediators, but that their timely resolution is surely crucial for resolving inflammation and inhibiting further damage [61]. Thus, NETosis and neutrophil degranulation need to be carefully regulated if post-MI wound healing is to proceed in a balanced way.

#### Proliferation and apoptosis of resident macrophages

Macrophages both induce inflammation and maintain homeostasis. These immune cells reside in the heart and account for about 5–10% of non-myocyte cells during steady state [66]. Following an ischemic injury, these numbers inflate due to an influx of macrophages from the circulatory system. For this reason, studies of macrophages in this context have mostly focused on their morphological and functional changes upon infiltrating damaged tissue. The roles and origins of resident cardiac macrophages have received less attention.

Tissue resident macrophages mostly develop embryonically, either from yolk sac (YS) or fetal liver haematopoietic stem cells (HSC) [57]. Once having taken up residence in the tissue, their turnover is tissue specific and tightly depends on the particular organ system [163]. In the heart, tissue macrophages renew mostly from local sources. Following their depletion in a mouse model, resident macrophages undergo an increased rate of local proliferation, based on depletion of the cells and staining for the cell cycle marker Ki67. This indicates a highly regenerative phenotype [44, 66]. But a study from Molawi et al. has suggested that newly recruited macrophages play a role in the turnover of resident cells, whose rate of self-proliferation gradually decreases with aging. They are replenished by monocyte derived macrophages, in contrast to previous reports saying that the regulation of resident macrophages is independent of those that circulate [44, 126]. A recent study by Bajpai et al. has characterized distinctive subsets of macrophage in the heart: CCR2^−^ tissue-resident macrophages and CCR2^+^ monocyte-derived macrophages. CCR2^−^ macrophages are maintained through self-proliferation, while CCR2^+^ indicates macrophages replenished by the recruitment of monocytes [9]. This heterogeneity may be explained by complex processes of modulation affecting the macrophage population in the tissue.

Cardiac-resident macrophages have functions distinct from the recruited macrophages [44]. These functions have recently attracted attention due to new findings on interactions between the immune cells, cardiomyocytes and endothelial cells [51]. A recent study from Nahrendorf et al. revealed that resident cardiac macrophages play an important role in maintaining the steady state in healthy mice. His group discovered that AV nodal macrophages are directly attached to cardiomyocytes via connexin-43 and contribute to steady-state electrical conduction in the heart [75]. How these functions change after cardiac injuries like MI requires further study, but these findings introduce the novel concept that interactions between macrophages and cardiomyocytes might influence on electrical conduction of the heart after MI.

Tissue-resident macrophages have been previously considered as M0 state; however, an interesting study from Pinto et al. has shown that in the heart, these macrophages are more likely oriented toward a M2 phenotype, as seen in the expression of characteristic marker genes such as CD163 and Mrc1. A loss of the M2 phenotype in resident macrophages is associated with exaggerated cardiac inflammation [148]. Maintaining cardiac homeostasis might require establishing an anti-inflammatory phenotype for resident macrophages. How this concept could functionally affect macrophages recruited after injuries such as MI has yet to be explored.

Over the years, several studies have indicated that macrophages might undergo apoptosis after injury. Within 12 h after MI, significant increases in counts of TUNEL^+^MAC3^+^ cells have been shown in the infarcted heart [66]. An in vivo study from Timo’s group observed a complete disappearance of resident macrophages in the infarct within 1 day post-MI. An infiltration of circulating monocytes into the infarct led to a recuperation of macrophage numbers within 4 days post-MI [66]. In any case, macrophage turnover clearly plays an important role during inflammatory responses to MI. A recent study by Ishikawa aimed to improve infarct healing through a novel approach targeting apoptosis inhibitor of macrophage (AIM). This factor plays a role in the accumulation of macrophages in the injured area. An AIM depletion model exhibited reduced cardiac rupture with decreased inflammatory macrophages, suggesting that resident macrophages must undergo apoptosis to improve outcomes in cardiac healing [77]. Similar effects were seen in experiments depleting MafB, a transcription factor which has shown to be involved in myelomonocytic differentiation and AIM expression. Its loss had an inhibitory effect on atherogenesis by reducing AIM [64].

### Adaptive immunity and immune suppression

#### Tolerogenic DCs (tDCs)

DC maturation can be divided into three phenotypes: immature, intermediated (semi-mature) and fully mature [109]. An increasingly popular type of immune therapy involves establishing DC tolerance mechanisms in an approach called tolerogenic therapy; it is being applied to a number of disease models including rheumatoid arthritis (RA) and sclerosis [119, 167]. While fully mature DCs are immunogenic, activating T-cell immunity, semi-mature DCs (tDCs) are tolerogenic, maintaining tolerance of T cells [101, 115, 178]. tDCs significantly upregulate their production of the well-known immune suppressor IL-10. This increases numbers of Tregs and suppresses immune responses [128, 195]. Treatment with tDCs induces macrophages to acquire a reparative phenotype by increasing the population of Tregs, preserving post-MI systolic LV functions and improving survival [24]. Zhu et al. showed that stimulating DCs with IL-37 and Troponin I can foster a tolerogenic phenotype. Such induced tDCs play a protective role in post-MI cardiac remodeling by suppressing Th1- or Th17-mediated inflammation [226]. These results suggest that tDC treatment might improve the immune environment as a novel therapeutic strategy for MI.

#### T-cell activation

T lymphocytes act as important regulators of adaptive immunity by inducing cell-mediated immune responses [158]. Activated T cells have been detected in both the infarct and remote areas of the heart tissue in patients with MI [1]. All types of T cells gradually infiltrate into the infarct in mice with permanent occlusion, with numbers peaking at 7 days post-MI [212]. The types can generally be distinguished with two categories: T helper (Th) cells and cytotoxic T (Tc) cells, on the basis of glycoproteins presented on the cell surface. Mature Th cells express CD4 on their surfaces and are referred to as a “helper CD4^+^ T cells”, while mature cells expressing CD8^+^ are known as “cytotoxic CD8^+^ T cells” [95]. CD4^+^ T cells are required for an immune response through their facilitation of the production of antibodies from B cells. They are involved in the regulation of inflammatory responses induced by macrophages and the recruitment of other immune cells to the injured sites. They also assist in CD8^+^ T-cell activation [81, 223]. Although their numbers are estimated to peak at < 1% of the total population of cardiac cells, CD4^+^ T-cell recruitment is significantly increased overall following an Ischemic insult, as determined by the enlargement of heart draining lymph nodes and an elevation in the total number of cells they contain in a permanently occluded mice model [69]. Cytotoxic CD8^+^ T cells, on the other hand, have the capacity to kill damaged or cancerous cells. Interestingly, the CD4/CD8 ratio has been found to be lower in patients with acute MI [173]. This trend is even more pronounced in MI patients who also suffer from HIV infections or Type 1 diabetes, in whom this significantly decreased CD4/CD8 ratio is associated with a higher post-MI mortality rate [8, 174].

#### CD4^+^ T-cell differentiation

CD4^+^ T cells can likewise be differentiated into distinct subtypes which play specific roles during the inflammatory response: Th1 (CD3^+^CD4^+^IFN-γ^+^), Th2 (CD3^+^CD4^+^ IL-4^+^), Th17 (CD3^+^CD4^+^IL-17A^+^) and regulatory T cells (Treg; CD4^+^CD25^+^Foxp3^+^) [224] (Table 1). The quantities of all types of T cells and B cells increase in the infarcted myocardium after MI, although levels of Th17 and Treg remain relatively low [188] (Fig. 1). Significantly higher Th1/Th2 and Th17/Treg ratios have been shown in particular patients with heart failure (CHF), compared to a healthy control. This change is associated with a heightened myocardial inflammation and indicates a strong relationship between the CD4^+^ T-cell-mediated immune response and cardiac function [17, 21].

Th1 cells mediate cellular immune responses and induce a pro-inflammatory reaction by producing TNF-α and IFN-γ. A complementary inhibition of Th1 activation improves post-MI LV wound healing [213]. Th2 cells mediate the humoral immune response (also referred to as the antibody-mediated response) and induce an anti-inflammatory reaction that is facilitated by a secretion of IL-4 and IL-13. High numbers of Th2 cells are associated with a lower risk of getting MI by preventing cell apoptosis [43]. Th17 cells mediate a strong pro-inflammatory response through the production of IL-17 through means resembling the activity of Th1 cells. The sustained production of IL-17 by Th17 increases inflammations, which suggests that Th17 has detrimental effects during post-MI cardiac repair [168]. Tregs are a subset of suppressor T cells expressing CD25 and Foxp3 and are important in balancing the immune response by regulating inflammation; they also provide tolerance to self Ags [224]. A depletion of Treg cells leads to an increase in LV dilation and a high rupture rate, and is associated with an acceleration of the post-MI infiltration of inflammatory cells [69]. Overall, methods that maintain better ratios between populations of Th1 × Th17 and Th2 × Tregs might offer potential therapeutic approaches to improve post-MI healing.

#### Treg-mediated immune suppression

Following MI, CD4^+^CD25^+^Foxp3^+^ Treg significantly accumulate in the region of the infarct, but their immunosuppressive functions are impaired [160]. Despite this, exogenous post-MI Treg treatment results in a reduction of infarct size and improved cardiac function, indicating that the transfer of Treg cells could be a novel therapeutic approach [160]. There is mounting evidence that Tregs play a crucial role in inflammatory resolution during post-MI remodeling. A study from Weirather’s group showed that a treatment with Tregs improved post-MI LV remodeling by regulating the phenotype of macrophages and enhanced the resolution of inflammation, further indicating a therapeutic potential of Tregs in the MI setting [206]. The beneficial effects of CD4^+^Foxp3^+^Tregs do not apply, however, to post-ischemia reperfusion (IR) injury. The injury is enhanced because during reperfusion, CD4^+^Foxp3^+^ T cells are immediately activated by either T-cell receptor (TCR)-independent signaling or preceding auto-antigen recognition. Although this process eventually leads to classical resolution of the inflammation by Foxp3^+^ Tregs, this means that care must be taken in clinical approaches based on mediating the activation of injurious T-cell subsets [121].

#### B cells

B lymphocytes mediate the humoral immune response by producing antibodies. The maturation of B cells occurs in the bone marrow, whereas activation occurs via B-cell receptors (BCRs) in secondary lymphoid organs. An important role in CD3^−^CD19^+^ cell activation is played by interactions between surface receptors (CD21) and surface proteins (CD19 and CD81). In mice, B cells accumulate in the infarct after permanent coronary ligation and their numbers peak 5–7 days post-MI [212, 230]. Following MI, mature B cells selectively release CCL7, whose abundance in the circulation is strongly correlated with high post-MI mortality rates. B-cell-induced CCL7 production contributes to impaired cardiac function by elevating monocyte recruitment into the infarct. Accordingly, B-cell depletion reduces the inflammatory response, emphasizing the need for a better understanding of the effects of B-cell suppression on MI healing [230].

## Summary: non-coding RNAs and immune regulation

Mounting evidence regarding the many diverse ways noncoding RNAs (ncRNAs) serve as master regulators of gene expression in diverse situations involving immunity and wound healing have brought these molecules to attention as potential targets for therapies. Studies have shown that ncRNAs are expressed in a highly lineage-specific manner and regulate the differentiation and function of innate and adaptive immune cells—both of which are crucial in attempts to develop therapies that target pathological processes with high specificity in the environment of post-MI cardiac tissues. A global disruption of immune cell types would almost invariably have negative consequences on patient health, particularly in the context of a dynamic tissue in which the roles of inflammatory and adaptive immune cells change over time.

A recent study from Halade et al. showed that miRNAs play a role in regulating gene expression related to leukocyte kinetics following MI. This suggests that modulating the MI-coordinated miRs could provide hints towards the regulation of post-MI inflammatory responses [62]. Wang et al. have reported a cardio-protective role for miR-146a. Its transfection inhibited the activation of NF-kB and diminished the infiltration of neutrophils into the heart following myocardial I/R, leading to reduced infarct size and improved cardiac function following MI [201]. Lowering the expression of another miRNA, miR-223, was associated with an increase in neutrophil infiltration and myocardial dysfunction in a sepsis patient via activation of STAT3/IL6 and Sema3, indicating that the presence of the miR-223 prevents this influx and lowers inflammation [202]. miR-21 and -150 also help prevent adverse MI remodeling through their effects on leukocyte numbers and subsequent vascular inflammation [11, 18, 113]. Deficiency of miR-21 in macrophages promotes apoptosis-related signaling pathways, including the MKK3/p38 and JNK pathways, leading to apoptosis and vascular inflammation during atherogenesis [18]. miR-150 negatively regulates expression of the chemokine receptor 4 (CXCR4) which in turn induces monocyte migration, thereby decreasing infiltration of inflammatory monocytes and improving cardiac function, as shown in miR-150 overexpressing mice [113]. A significantly reduced expression of miR-144 is associated with improper cardiac remodeling, while restoring endogenous levels of myocardial expression of miR-144 through intravenous injections improves post-MI cardiac function. Additional mechanistic studies have demonstrated that miR-144 inhibits inflammatory and auto-phagocytic signaling pathways, indicating that miR-144 might have its beneficial effects by lowering the infiltration of macrophages and improving autophagy [106].

MiR-155 expression is significantly and primarily upregulated in macrophages, in the post-MI myocardium; interestingly, levels differ between M1 and M2 macrophages. Its depletion promotes M2 polarization and improves cardiac function following viral myocarditis [219]. This suggests that miRNA-155 might serve as a prognostic marker for cardiac death in post-MI patients [123]. miR-155 is also found in exosomes released by macrophages and this has effects on fibroblasts, which in turn trigger a dysregulation of fibrosis [197]. miR-155 is mainly involved in B- and T-cell receptor signaling, neurotrophin signaling, MAPK signaling, and the cell cycle. Especially in regard to the cell-cycle signaling pathway, Sos1 expression is increased in the absence of miR-155, and is associated with fibroblast proliferation post-MI [197]. Also, angiotensin II-induced expression of the angiotensin II type 1 receptor (AT1R) and extracellular signal-related kinase 1/2 (ERK1/2) are downregulated by miR-155 [123]. Overall, the inhibition of miR-155 activity seems to have therapeutic potential in seeking to minimize post-MI cardiac injury [197].

Circulating miR-133 and miR-33 directly promotes macrophage polarization and has further effects on lipid metabolism, as seen in the myocardial steatosis that develops in type 2 diabetes patients [34, 143]. miR-33 expression levels have been used as diagnostic marker for diabetic cardiomyopathy [34]. miR-33 mediates anti-inflammatory macrophage polarization by targeting the energy sensor AMP-activated protein kinase (AMPK) pathway [143]. Two further miRNAs, miR-150 and miR-181a, play roles in regulating both DC differentiation and vascular inflammation. Necrotic cardiomyocyte-stimulated DC maturation requires the JAK1-STAT1/c-Fos pathway, concomitant with decreased miR-150 and increased miR-181a levels. Modification of these miRNAs, either through the overexpression of miR-150 or through the inhibition of miR-181a, respectively, downregulates DC maturation and leads to a reduction in the apoptosis of cardiomyocytes, indicating a potential therapeutic approach to preserve cardiomyocytes after a cardiac injury such as MI [225].

Various lncRNAs have also been implicated in immune regulation (Table 2). Vausort et al. showed a strong connection between the inflammatory response and lncRNAs including hypoxia inducible factor 1A antisense RNA 2 (aHIF), cyclin-dependent kinase inhibitor 2B antisense RNA 1 (ANRIL), MI-associated transcript (MIAT) and metastasis-associated lung adenocarcinoma transcript 1 (MALAT1). Their expression was closely associated with blood cell count as well as the abundance of neutrophils and lymphocytes. Cardiovascular risk factors such as aging or diabetes boost their levels even higher. So far, a direct connection to post-MI cardiac dysfunction has only been established for ANRIL [190]. Levels of the myocardial infarction-associated transcript-1(Mirt1) and Mirt2 are elevated in MI and peak at 24 h post-MI, strongly suggesting another case of ncRNAs linked to inflammatory regulation [215]. LPS-induced Mirt2 regulates inflammatory cytokine production through the polarization of macrophages towards a M2 phenotype via suppression of NF-κB and MAPK pathways [39]. Mirt2 overexpression protects against endotoxemia-induced mortality and organ dysfunction [39]. Furthermore, both Mirt1 and Mirt2 target cardiac remodeling genes such as mmp-9, Icam1 and tgfb1 during post-MI wound healing [215]. The upregulation of both lncRNAs have been negatively correlated with post-MI cardiac remodeling, accompanied by the smaller size of infarcts and better ejection fractions, indicating that elevations in Mirt1 and Mirt2 expression balance the inflammatory response and preserve cardiac function post-MI [215].

**Table 2:** Non-coding RNAs as a biomarker and therapeutic approaches in myocardial infarction

Therapeutic approaches	Non-coding RNA	RNA name	Functions	Major cell source	Post-MI level
Inhibition of inflammation	miRNA	miR-144	↓Pro-inflammatory response	N/A	↓
		miR-146a	↓Infiltration of neutrophils, infarct size	Cardiomyocytes	↑
		miR-150, -181a	↓DCs maturation, cardiomyocyte apoptosis	Dendritic cells	↑
		miR-223	↑Neutrophil infiltration, inflammation	Cardiac muscles	↓
		Let-7i-5p	↓Inflammatory cytokine production, fibrosis	Fibroblasts	↑
	lncRNA	ANRIL	Increase blood cell count, associated with cardiac dysfunction	PBMCs	↑
		LINC00305	Accelerated monocyte-mediated inflammation	PBMCs	↑
		LncRNA-1055, -A930015D03Rik	↑Th1 mediated immune response, ↑cardiac inflammation	N/A	N/A
Resolution of inflammation	miRNA	miR-133	Pro-inflammatory macrophage polarization, lipid metabolism	Macrophages	↑
		miR-155	Macrophage polarization, ↑fibroblast proliferation ↑Treg proliferation	Macrophages	↑
	lncRNA	Mirt1, -2	Macrophage polarization, ↑cardiac function	Fibroblasts	↑
Modulation adoptive immunity	lncRNA	LncRNA-E330013P06	↑Foam cell production, ↑atherosclerosis	Macrophage	↑

*LncRNA (lnc-)* long non coding RNA, *miRNA (mir-)* microRNA, *PBMC* peripheral blood mononuclear cells, *DC* dendritic cells

The inflammatory response post-MI can result in systemic atherosclerosis with elevated numbers of macrophage-derived foam cells accompanied by enhanced lipid metabolism [85]. LncRNA E330013P06 is expressed at high levels in foam cells, a phenomenon associated with exaggerated cardiac inflammation. Sustained E330013P06 levels are correlated with elevated levels of pro-inflammatory genes and pro-atherogenic genes, which contribute to foam cell formation. Inhibiting the expression of E330013P06 in macrophages reduces the production of both foam cells and the expression of inflammatory genes in diabetes patients [152]. There are still crucial questions regarding the E330013P06 underlying molecular mechanism that controls the cardiac inflammatory response, which merits further studies. A further pro-inflammatory lncRNA, LINC00305, is also highly expressed in monocytes derived from patients with atherosclerosis and is associated with an exaggerated inflammatory response [217]. LINC00305 promotes an interaction between the membrane protein lipocalin-interacting membrane receptor (LIMR) and the inflammatory gene aryl hydrocarbon receptor repressor (AHRR) via activation of the NF-kappaB pathway in human monocytes, further contributing to the development of atherosclerosis [217]. These findings suggest that LINC00305 could be a novel target for an anti-inflammatory therapy. LncRNAs can also regulate autoimmunity, as the expression of lncRNA-A930015D03Rik and -1055 is strongly correlated with IL12Rβ1, one of the essential molecular markers in Th1 response pathway. Knocking down lncRNA-A930015D03Rik and -1055 to modulate the Th1-mediated immune response and cardiac inflammation is an interesting line of future therapeutic strategies [56].

A function in the context of immunity has not been described for most ncRNAs, although dramatic changes in ncRNA expression have been clearly shown during the activation of immune cells. This further strengthens the argument that ncRNAs can act as immune regulators and should therefore not be considered mere transcriptional ‘noise’. Further investigations into ncRNAs and their potential immune functions will undoubtedly yield insights into the mechanisms that balance the inflammatory response, which could ultimately lead to improved treatments for cardiovascular diseases and other pathologies.

## Non-coding RNA based therapeutics

An increasing number and range of functions are being found for ncRNAs in processes related to the dynamic development, function and activation of cells, all of which are relevant to pathologies. This has given rise to the concept of manipulating disease-related signaling pathways by targeting cell-specific ncRNAs in developing new approaches to therapies. The main aim of such ncRNA-based therapies has generally been to alter abnormal levels of expression of ncRNAs by restoring them to the basal level. In the investigation of ncRNA functions, antagonists and ncRNA mimics are being effectively used; most therapeutic efforts are based on these strategies as well. [154]. Antisense technologies which sequester or degrade mature ncRNAs are currently the most efficient approaches in silencing ncRNA activity.

miRNAs seem to be particularly targetable through the delivery of reverse complimentary anti-miRNA oligonucleotides, which function by either sequestering the target molecule or by triggering their degradation by cellular RNA interference mechanisms. Antagonists usually need to be modified to enhance their stability and improve binding efficiency, through chemical modifications such as the addition of 2-*O*-methyl groups, or methylene linkers that ‘lock’ the oligonucleotides in a more robust conformation (LNA) [179]. Additionally, cholesterol-conjugated antagomirs might be useful tools, as demonstrated by Krutzfeldt et al. who achieved a cardiac tissue-specific miRNA knockdown by injecting the compounds into mice [98]. The LNA-based approach is currently being tested in an anti-miRNA therapy in stage IIa of clinical trials, using an inhibitor designed to target miR-122 in cases of chronic Hepatitis C infection. This study represents a good example how to translate a miRNA-focused therapy in the clinic, and it suggests that targeting immune cell-derived miRNAs in the heart is a practicable strategy.

Another concept that has emerged in altering miRNA expression involves “sponges”, which do not actively trigger their degradation but rather serve as baits that prevent their binding to target mRNAs. Such sponges may be constructed to target multiple miRNAs and have longer lifespans than miRNA inhibitors. Wang’s group has designed a ‘Multiple Target AMO Technology (MT-AMO)’, in the form of a single-stranded, methyl-modified oligonucleotide sequence capable of binding multiple miRNAs within a single family of seeds or even multiple families [203]. This could be useful in treating human pathologies, including cardiovascular diseases, in which several miRNAs are deregulated.

The novel CRISPR/cas9 system has also been used to knock-down expression of ncRNAs [20, 26]. Chang et al. demonstrated that the CRISPR approach not only reduces the off-target seen with miR inhibitors or mimics, but also has a knock-down effect on miRNAs that is sustained much longer in both in vitro and in vivo models [20]. Synthetic ncRNAs and individual ncRNAs transduced using viruses are the most common method of restoring levels of downregulated ncRNAs [194], but the results are not always reproducible or very potent, making efforts to re-constitute miRNA levels lag behind those aimed at depleting them.

Our review shows that levels of expression of an increasing number of ncRNAs are now known to change following MI [190]. Recent work has deepened our understanding of the way the immune regulation of non-coding RNAs influence post-MI cardiac functions, particularly where the inflammatory response is concerned. The findings open ncRNAs to new approaches for clinical translation in efforts to achieve optimal post-MI wound healing.

## Conclusion and future direction

Dynamic immune responses regulate key events during post-MI cardiac repair. Distinct types of immune cells act in precisely timed ways and take on diverse roles in preserving cardiac function after MI. Improper or imbalanced immune responses have adverse effects on LV remodeling and enhance the progression to heart failure. Understanding these cell fates and functions and the diverse factors related to immune action will be required to help develop an improved microenvironment that encourages repair. A number of approaches to regulate the immune response following MI damage are under development. The first step in moving these concepts into clinical practice is to understand how ncRNAs regulate the functions of the immune cell repertoire in achieving a balanced inflammatory response in the context of the post-MI heart. Given that key molecules are expressed in different cell types, often with contradictory functions at different time points, applications will require a very profound understanding of the cell-specific functions of ncRNAs and the way they change over time.

Data are particularly needed on the way the origin and spatiotemporal distribution of these immune cells function in the broader context of immunity, which has not been documented here. To date, most experimental models have been limited because they are based on models where age, gender and genetic background are highly standardized. This is never sufficient in characterizing the progression of diseases that affect patients who are mostly older and have co-morbidities [141]. What will be needed is a “higher resolution” view of the kinetics of immune cells over time in different strains of animals, particularly during dynamic inflammatory states in areas of the heart and body beyond those directly affected by an infarction. While much has been learned about the dynamics of immune cells that closely associated with cardiac dysfunction in the ischemic myocardium, much less is known about inflammatory changes in remote post-MI myocardium. There is evidence that remote myocardium dysfunction indirectly or directly contributes to functional and morphological changes in the infarct region [15]. To thoroughly understand the systemic immune response following MI, further studies will be needed that focus not only on ischemic lesions, but also on non-ischemic lesions; they will need to be carried out in a wider range of experimental models with some connection to cardiovascular physiology. Another issue will be to characterize the crosstalk between immune cells, other cardiac cells and those in other tissues. There is every sign that addressing these gaps in our knowledge will identify fruitful new avenues toward diagnosing, treating and preventing MI, as a means of improving the lives of the growing number of patients suffering from this dreaded disease.

## References

[1] Abbate A, Bonanno E, Mauriello A, Bussani R, Biondi-Zoccai GG, Liuzzo G, Leone AM, Silvestri F, Dobrina A, Baldi F, Pandolfi F, Biasucci LM, Baldi A, Spagnoli LG, Crea F (2004). Widespread myocardial inflammation and infarct-related artery patency. Circulation.

[2] Ali M, Pulli B, Courties G, Tricot B, Sebas M, Iwamoto Y, Hilgendorf I, Schob S, Dong A, Zheng W, Skoura A, Kalgukar A, Cortes C, Ruggeri R, Swirski FK, Nahrendorf M, Buckbinder L, Chen JW (2016). Myeloperoxidase inhibition improves ventricular function and remodeling after experimental myocardial infarction. JACC Basic Transl Sci.

[3] Antonatos D, Patsilinakos S, Spanodimos S, Korkonikitas P, Tsigas D (2006). Cell-free DNA levels as a prognostic marker in acute myocardial infarction. Ann N Y Acad Sci.

[4] Antoniucci D (2014). Block the ischemia and reperfusion damage: an old adjunctive drug for a new reperfusion strategy. J Am Coll Cardiol.

[5] Aoki S, Nakagomi A, Asai K, Takano H, Yasutake M, Seino Y, Mizuno K (2011). Elevated peripheral blood mononuclear cell count is an independent predictor of left ventricular remodeling in patients with acute myocardial infarction. J Cardiol.

[6] Aune TM, Crooke PS, Spurlock CF (2016). Long noncoding RNAs in T lymphocytes. J Leukoc Biol.

[7] Backteman K, Ernerudh J, Jonasson L (2014). Natural killer (NK) cell deficit in coronary artery disease: no aberrations in phenotype but sustained reduction of NK cells is associated with low-grade inflammation. Clin Exp Immunol.

[8] Badejo OA, Chang CC, So-Armah KA, Tracy RP, Baker JV, Rimland D, Butt AA, Gordon AJ, Rinaldo CR, Kraemer K, Samet JH, Tindle HA, Goetz MB, Rodriguez-Barradas MC, Bedimo R, Gibert CL, Leaf DA, Kuller LH, Deeks SG, Justice AC, Freiberg MS (2015). CD8^+^T-cells count in acute myocardial infarction in HIV disease in a predominantly male cohort. Biomed Res Int.

[9] Bajpai G, Schneider C, Wong N, Bredemeyer A, Hulsmans M, Nahrendorf M, Epelman S, Kreisel D, Liu Y, Itoh A, Shankar TS, Selzman CH, Drakos SG, Lavine KJ (2018). The human heart contains distinct macrophage subsets with divergent origins and functions. Nat Med.

[10] Banchereau J, Briere F, Caux C, Davoust J, Lebecque S, Liu YJ, Pulendran B, Palucka K (2000). Immunobiology of dendritic cells. Annu Rev Immunol.

[11] Barnett RE, Conklin DJ, Ryan L, Keskey RC, Ramjee V, Sepulveda EA, Srivastava S, Bhatnagar A, Cheadle WG (2016). Anti-inflammatory effects of miR-21 in the macrophage response to peritonitis. J Leukoc Biol.

[12] Bazzoni F, Rossato M, Fabbri M, Gaudiosi D, Mirolo M, Mori L, Tamassia N, Mantovani A, Cassatella MA, Locati M (2009). Induction and regulatory function of miR-9 in human monocytes and neutrophils exposed to proinflammatory signals. Proc Natl Acad Sci USA.

[13] Blum A, Yeganeh S (2003). The role of T-lymphocyte subpopulations in acute myocardial infarction. Eur J Intern Med.

[14] Bluml S, Bonelli M, Niederreiter B, Puchner A, Mayr G, Hayer S, Koenders MI, van den Berg WB, Smolen J, Redlich K (2011). Essential role of microRNA-155 in the pathogenesis of autoimmune arthritis in mice. Arthritis Rheum.

[15] Bogaert J, Bosmans H, Maes A, Suetens P, Marchal G, Rademakers FE (2000). Remote myocardial dysfunction after acute anterior myocardial infarction: impact of left ventricular shape on regional function: a magnetic resonance myocardial tagging study. J Am Coll Cardiol.

[16] Bratton DL, Henson PM (2011). Neutrophil clearance: when the party is over, clean-up begins. Trends Immunol.

[17] Cai YH, Ma ZJ, Lu XY, He EL, You MY (2016). Study on the effect and mechanism of the dysfunction of CD4(+) T cells in the disease process of chronic cardiac failure. Asian Pac J Trop Med.

[18] Canfran-Duque A, Rotllan N, Zhang X, Fernandez-Fuertes M, Ramirez-Hidalgo C, Araldi E, Daimiel L, Busto R, Fernandez-Hernando C, Suarez Y (2017). Macrophage deficiency of miR-21 promotes apoptosis, plaque necrosis, and vascular inflammation during atherogenesis. EMBO Mol Med.

[19] Chan W, White DA, Wang XY, Bai RF, Liu Y, Yu HY, Zhang YY, Fan F, Schneider HG, Duffy SJ, Taylor AJ, Du XJ, Gao W, Gao XM, Dart AM (2013). Macrophage migration inhibitory factor for the early prediction of infarct size. J Am Heart Assoc.

[20] Chang H, Yi B, Ma R, Zhang X, Zhao H, Xi Y (2016). CRISPR/cas9, a novel genomic tool to knock down microRNA in vitro and in vivo. Sci Rep.

[21] Cheng X, Liao YH, Ge H, Li B, Zhang J, Yuan J, Wang M, Liu Y, Guo Z, Chen J, Zhang J, Zhang L (2005). TH1/TH2 functional imbalance after acute myocardial infarction: coronary arterial inflammation or myocardial inflammation. J Clin Immunol.

[22] Cheng Y, Rong J (2017). Macrophage polarization as a therapeutic target in myocardial infarction. Curr Drug Targets.

[23] Chia S, Nagurney JT, Brown DF, Raffel OC, Bamberg F, Senatore F, Wackers FJ, Jang IK (2009). Association of leukocyte and neutrophil counts with infarct size, left ventricular function and outcomes after percutaneous coronary intervention for ST-elevation myocardial infarction. Am J Cardiol.

[24] Choo EH, Lee JH, Park EH, Park HE, Jung NC, Kim TH, Koh YS, Kim E, Seung KB, Park C, Hong KS, Kang K, Song JY, Seo HG, Lim DS, Chang K (2017). Infarcted myocardium-primed dendritic cells improve remodeling and cardiac function after myocardial infarction by modulating the regulatory T cell and macrophage polarization. Circulation.

[25] Courties G, Heidt T, Sebas M, Iwamoto Y, Jeon D, Truelove J, Tricot B, Wojtkiewicz G, Dutta P, Sager HB, Borodovsky A, Novobrantseva T, Klebanov B, Fitzgerald K, Anderson DG, Libby P, Swirski FK, Weissleder R, Nahrendorf M (2014). In vivo silencing of the transcription factor IRF5 reprograms the macrophage phenotype and improves infarct healing. J Am Coll Cardiol.

[26] Covarrubias S, Robinson EK, Shapleigh B, Vollmers A, Katzman S, Hanley N, Fong N, McManus MT, Carpenter S (2017). CRISPR/Cas-based screening of long non-coding RNAs (lncRNAs) in macrophages with an NF-kappaB reporter. J Biol Chem.

[27] Cramer T, Yamanishi Y, Clausen BE, Forster I, Pawlinski R, Mackman N, Haase VH, Jaenisch R, Corr M, Nizet V, Firestein GS, Gerber HP, Ferrara N, Johnson RS (2003). HIF-1alpha is essential for myeloid cell-mediated inflammation. Cell.

[28] Cross A, Moots RJ, Edwards SW (2008). The dual effects of TNFalpha on neutrophil apoptosis are mediated via differential effects on expression of Mcl-1 and Bfl-1. Blood.

[29] Cui BB, Tan CY, Schorn C, Tang HH, Liu Y, Zhao Y (2012). Neutrophil extracellular traps in sterile inflammation: the story after dying?. Autoimmunity.

[30] Cui H, Xie N, Tan Z, Banerjee S, Thannickal VJ, Abraham E, Liu G (2014). The human long noncoding RNA lnc-IL7R regulates the inflammatory response. Eur J Immunol.

[31] Cui M, Fan M, Jing R, Wang H, Qin J, Sheng H, Wang Y, Wu X, Zhang L, Zhu J, Ju S (2013). Cell-Free circulating DNA: a new biomarker for the acute coronary syndrome. Cardiology.

[32] Curley D, Lavin Plaza B, Shah AM, Botnar RM (2018) Molecular imaging of cardiac remodelling after myocardial infarction. Basic Res Cardiol 113:10-018-0668-z 10.1007/s00395-018-0668-z 10.1007/s00395-018-0668-z PMC5772148 29344827

[33] Curtsinger JM, Mescher MF (2010). Inflammatory cytokines as a third signal for T cell activation. Curr Opin Immunol.

[34] de Gonzalo-Calvo D, van der Meer RW, Rijzewijk LJ, Smit JW, Revuelta-Lopez E, Nasarre L, Escola-Gil JC, Lamb HJ, Llorente-Cortes V (2017). Serum microRNA-1 and microRNA-133a levels reflect myocardial steatosis in uncomplicated type 2 diabetes. Sci Rep.

[35] DeLeon-Pennell KY, Tian Y, Zhang B, Cates CA, Iyer RP, Cannon P, Shah P, Aiyetan P, Halade GV, Ma Y, Flynn E, Zhang Z, Jin YF, Zhang H, Lindsey ML (2016). CD36 is a matrix metalloproteinase-9 substrate that stimulates neutrophil apoptosis and removal during cardiac remodeling. Circ Cardiovasc Genet.

[36] Delgado-Martin C, Escribano C, Pablos JL, Riol-Blanco L, Rodriguez-Fernandez JL (2011). Chemokine CXCL12 uses CXCR4 and a signaling core formed by bifunctional Akt, extracellular signal-regulated kinase (ERK)1/2, and mammalian target of rapamycin complex 1 (mTORC1) proteins to control chemotaxis and survival simultaneously in mature dendritic cells. J Biol Chem.

[37] Dieterlen MT, John K, Reichenspurner H, Mohr FW, Barten MJ (2016). Dendritic cells and their role in cardiovascular diseases: a view on human studies. J Immunol Res.

[38] Domingo-Gonzalez R, Martinez-Colon GJ, Smith AJ, Smith CK, Ballinger MN, Xia M, Murray S, Kaplan MJ, Yanik GA, Moore BB (2016). Inhibition of neutrophil extracellular trap formation after stem cell transplant by prostaglandin E2. Am J Respir Crit Care Med.

[39] Du M, Yuan L, Tan X, Huang D, Wang X, Zheng Z, Mao X, Li X, Yang L, Huang K, Zhang F, Wang Y, Luo X, Huang D, Huang K (2017). The LPS-inducible lncRNA Mirt2 is a negative regulator of inflammation. Nat Commun.

[40] Dueck A, Eichner A, Sixt M, Meister G (2014). A miR-155-dependent microRNA hierarchy in dendritic cell maturation and macrophage activation. FEBS Lett.

[41] Dunand-Sauthier I, Santiago-Raber ML, Capponi L, Vejnar CE, Schaad O, Irla M, Seguin-Estevez Q, Descombes P, Zdobnov EM, Acha-Orbea H, Reith W (2011). Silencing of c-Fos expression by microRNA-155 is critical for dendritic cell maturation and function. Blood.

[42] Dutta P, Nahrendorf M (2015). Monocytes in myocardial infarction. Arterioscler Thromb Vasc Biol.

[43] Engelbertsen D, Andersson L, Ljungcrantz I, Wigren M, Hedblad B, Nilsson J, Bjorkbacka H (2013). T-helper 2 immunity is associated with reduced risk of myocardial infarction and stroke. Arterioscler Thromb Vasc Biol.

[44] Epelman S, Lavine KJ, Beaudin AE, Sojka DK, Carrero JA, Calderon B, Brija T, Gautier EL, Ivanov S, Satpathy AT, Schilling JD, Schwendener R, Sergin I, Razani B, Forsberg EC, Yokoyama WM, Unanue ER, Colonna M, Randolph GJ, Mann DL (2014). Embryonic and adult-derived resident cardiac macrophages are maintained through distinct mechanisms at steady state and during inflammation. Immunity.

[45] Fadok VA, Bratton DL, Konowal A, Freed PW, Westcott JY, Henson PM (1998). Macrophages that have ingested apoptotic cells in vitro inhibit proinflammatory cytokine production through autocrine/paracrine mechanisms involving TGF-beta, PGE2, and PAF. J Clin Invest.

[46] Fang L, Du XJ, Gao XM, Dart AM (2010). Activation of peripheral blood mononuclear cells and extracellular matrix and inflammatory gene profile in acute myocardial infarction. Clin Sci (Lond).

[47] Fang L, Moore XL, Dart AM, Wang LM (2015). Systemic inflammatory response following acute myocardial infarction. J Geriatr Cardiol.

[48] Frangogiannis NG (2012). Regulation of the inflammatory response in cardiac repair. Circ Res.

[49] Frantz S, Bauersachs J, Ertl G (2009). Post-infarct remodelling: contribution of wound healing and inflammation. Cardiovasc Res.

[50] Frodermann V, Nahrendorf M (2017). Neutrophil-macrophage cross-talk in acute myocardial infarction. Eur Heart J.

[51] Fujiu K, Wang J, Nagai R (2014). Cardioprotective function of cardiac macrophages. Cardiovasc Res.

[52] Gao XM, Liu Y, White D, Su Y, Drew BG, Bruce CR, Kiriazis H, Xu Q, Jennings N, Bobik A, Febbraio MA, Kingwell BA, Bucala R, Fingerle-Rowson G, Dart AM, Morand EF, Du XJ (2011). Deletion of macrophage migration inhibitory factor protects the heart from severe ischemia-reperfusion injury: a predominant role of anti-inflammation. J Mol Cell Cardiol.

[53] Garcia-Prieto J, Villena-Gutierrez R, Gomez M, Bernardo E, Pun-Garcia A, Garcia-Lunar I, Crainiciuc G, Fernandez-Jimenez R, Sreeramkumar V, Bourio-Martinez R, Garcia-Ruiz JM, Del Valle AS, Sanz-Rosa D, Pizarro G, Fernandez-Ortiz A, Hidalgo A, Fuster V, Ibanez B (2017). Neutrophil stunning by metoprolol reduces infarct size. Nat Commun.

[54] Ge L, Zhou X, Ji WJ, Lu RY, Zhang Y, Zhang YD, Ma YQ, Zhao JH, Li YM (2015). Neutrophil extracellular traps in ischemia-reperfusion injury-induced myocardial no-reflow: therapeutic potential of DNase-based reperfusion strategy. Am J Physiol Heart Circ Physiol.

[55] Gombozhapova A, Rogovskaya Y, Shurupov V, Rebenkova M, Kzhyshkowska J, Popov SV, Karpov RS, Ryabov V (2017). Macrophage activation and polarization in post-infarction cardiac remodeling. J Biomed Sci.

[56] Gomes CPC, Spencer H, Ford KL, Michel LYM, Baker AH, Emanueli C, Balligand JL, Devaux Y, Cardiolinc Network (2017). The function and therapeutic potential of long non-coding RNAs in cardiovascular development and disease. Mol Ther Nucleic Acids.

[57] Gomez Perdiguero E, Klapproth K, Schulz C, Busch K, Azzoni E, Crozet L, Garner H, Trouillet C, de Bruijn MF, Geissmann F, Rodewald HR (2015). Tissue-resident macrophages originate from yolk-sac-derived erythro-myeloid progenitors. Nature.

[58] Gordon S, Pluddemann A (2017). Tissue macrophages: heterogeneity and functions. BMC Biol.

[59] Gu H, Liu Z, Zhou L (2017). Roles of miR-17-92 cluster in cardiovascular development and common diseases. Biomed Res Int.

[60] Hahn J, Knopf J, Maueroder C, Kienhofer D, Leppkes M, Herrmann M (2016). Neutrophils and neutrophil extracellular traps orchestrate initiation and resolution of inflammation. Clin Exp Rheumatol.

[61] Halade GV, Kain V, Serhan CN (2018). Immune responsive resolvin D1 programs myocardial infarction-induced cardiorenal syndrome in heart failure. FASEB J.

[62] Halade GV, Norris PC, Kain V, Serhan CN, Ingle KA (2018). Splenic leukocytes define the resolution of inflammation in heart failure. Sci Signal.

[63] Hamada M, Nakamura M, Tran MT, Moriguchi T, Hong C, Ohsumi T, Dinh TT, Kusakabe M, Hattori M, Katsumata T, Arai S, Nakashima K, Kudo T, Kuroda E, Wu CH, Kao PH, Sakai M, Shimano H, Miyazaki T, Tontonoz P, Takahashi S (2014). MafB promotes atherosclerosis by inhibiting foam-cell apoptosis. Nat Commun.

[64] Harel-Adar T, Ben Mordechai T, Amsalem Y, Feinberg MS, Leor J, Cohen S (2011). Modulation of cardiac macrophages by phosphatidylserine-presenting liposomes improves infarct repair. Proc Natl Acad Sci USA.

[65] Heidt T, Courties G, Dutta P, Sager HB, Sebas M, Iwamoto Y, Sun Y, Da Silva N, Panizzi P, van der Laan AM, Swirski FK, Weissleder R, Nahrendorf M (2014). Differential contribution of monocytes to heart macrophages in steady-state and after myocardial infarction. Circ Res.

[66] Heusch G, Libby P, Gersh B, Yellon D, Bohm M, Lopaschuk G, Opie L (2014). Cardiovascular remodelling in coronary artery disease and heart failure. Lancet.

[67] Heward JA, Lindsay MA (2014). Long non-coding RNAs in the regulation of the immune response. Trends Immunol.

[68] Hofmann U, Beyersdorf N, Weirather J, Podolskaya A, Bauersachs J, Ertl G, Kerkau T, Frantz S (2012). Activation of CD4^+^ T lymphocytes improves wound healing and survival after experimental myocardial infarction in mice. Circulation.

[69] Hofmann U, Frantz S (2016). Role of T-cells in myocardial infarction. Eur Heart J.

[70] Horckmans M, Ring L, Duchene J, Santovito D, Schloss MJ, Drechsler M, Weber C, Soehnlein O, Steffens S (2017). Neutrophils orchestrate post-myocardial infarction healing by polarizing macrophages towards a reparative phenotype. Eur Heart J.

[71] Hsueh YC, Wu JM, Yu CK, Wu KK, Hsieh PC (2014). Prostaglandin E(2) promotes post-infarction cardiomyocyte replenishment by endogenous stem cells. EMBO Mol Med.

[72] Hu R, Kagele DA, Huffaker TB, Runtsch MC, Alexander M, Liu J, Bake E, Su W, Williams MA, Rao DS, Moller T, Garden GA, Round JL, O’Connell RM (2014). miR-155 promotes T follicular helper cell accumulation during chronic, low-grade inflammation. Immunity.

[73] Huang S, He X, Ding J, Liang L, Zhao Y, Zhang Z, Yao X, Pan Z, Zhang P, Li J, Wan D, Gu J (2008). Upregulation of miR-23a approximately 27a approximately 24 decreases transforming growth factor-beta-induced tumor-suppressive activities in human hepatocellular carcinoma cells. Int J Cancer.

[74] Hulsmans M, Clauss S, Xiao L, Aguirre AD, King KR, Hanley A, Hucker WJ, Wulfers EM, Seemann G, Courties G, Iwamoto Y, Sun Y, Savol AJ, Sager HB, Lavine KJ, Fishbein GA, Capen DE, Da Silva N, Miquerol L, Wakimoto H, Seidman CE, Seidman JG, Sadreyev RI, Naxerova K, Mitchell RN, Brown D, Libby P, Weissleder R, Swirski FK, Kohl P, Vinegoni C, Milan DJ, Ellinor PT, Nahrendorf M (2017). Macrophages facilitate electrical conduction in the heart. Cell.

[75] Hulsmans M, Holvoet P (2013). MicroRNA-containing microvesicles regulating inflammation in association with atherosclerotic disease. Cardiovasc Res.

[76] Ishikawa S, Noma T, Fu HY, Matsuzaki T, Ishizawa M, Ishikawa K, Murakami K, Nishimoto N, Nishiyama A, Minamino T (2017). Apoptosis inhibitor of macrophage depletion decreased M1 macrophage accumulation and the incidence of cardiac rupture after myocardial infarction in mice. PLoS One.

[77] Iyer RP, Patterson NL, Zouein FA, Ma Y, Dive V, de Castro Bras LE, Lindsey ML (2015). Early matrix metalloproteinase-12 inhibition worsens post-myocardial infarction cardiac dysfunction by delaying inflammation resolution. Int J Cardiol.

[78] Jadapalli JK, Halade GV (2018). Unified nexus of macrophages and maresins in cardiac reparative mechanisms. FASEB J.

[79] Jakubzick CV, Randolph GJ, Henson PM (2017). Monocyte differentiation and antigen-presenting functions. Nat Rev Immunol.

[80] Janssen EM, Lemmens EE, Wolfe T, Christen U, von Herrath MG, Schoenberger SP (2003). CD4^+^ T cells are required for secondary expansion and memory in CD8^+^ T lymphocytes. Nature.

[81] Jiang K, Sun X, Chen Y, Shen Y, Jarvis JN (2015). RNA sequencing from human neutrophils reveals distinct transcriptional differences associated with chronic inflammatory states. BMC Med Genom.

[82] Johnnidis JB, Harris MH, Wheeler RT, Stehling-Sun S, Lam MH, Kirak O, Brummelkamp TR, Fleming MD, Camargo FD (2008). Regulation of progenitor cell proliferation and granulocyte function by microRNA-223. Nature.

[83] Jonasson L, Backteman K, Ernerudh J (2005). Loss of natural killer cell activity in patients with coronary artery disease. Atherosclerosis.

[84] Joshi NV, Toor I, Shah AS, Carruthers K, Vesey AT, Alam SR, Sills A, Hoo TY, Melville AJ, Langlands SP, Jenkins WS, Uren NG, Mills NL, Fletcher AM, van Beek EJ, Rudd JH, Fox KA, Dweck MR, Newby DE (2015). Systemic atherosclerotic inflammation following acute myocardial infarction: myocardial infarction begets myocardial infarction. J Am Heart Assoc.

[85] Jung M, Ma Y, Iyer RP, DeLeon-Pennell KY, Yabluchanskiy A, Garrett MR, Lindsey ML (2017). IL-10 improves cardiac remodeling after myocardial infarction by stimulating M2 macrophage polarization and fibroblast activation. Basic Res Cardiol.

[86] Kaikita K, Hayasaki T, Okuma T, Kuziel WA, Ogawa H, Takeya M (2004). Targeted deletion of CC chemokine receptor 2 attenuates left ventricular remodeling after experimental myocardial infarction. Am J Pathol.

[87] Kain V, Prabhu SD, Halade GV (2014). Inflammation revisited: inflammation versus resolution of inflammation following myocardial infarction. Basic Res Cardiol.

[88] Kawaguchi M, Takahashi M, Hata T, Kashima Y, Usui F, Morimoto H, Izawa A, Takahashi Y, Masumoto J, Koyama J, Hongo M, Noda T, Nakayama J, Sagara J, Taniguchi S, Ikeda U (2011). Inflammasome activation of cardiac fibroblasts is essential for myocardial ischemia/reperfusion injury. Circulation.

[89] Kaya MG, Akpek M, Lam YY, Yarlioglues M, Celik T, Gunebakmaz O, Duran M, Ulucan S, Keser A, Oguzhan A, Gibson MC (2013). Prognostic value of neutrophil/lymphocyte ratio in patients with ST-elevated myocardial infarction undergoing primary coronary intervention: a prospective, multicenter study. Int J Cardiol.

[90] Kim D, Haynes CL (2012). Neutrophil chemotaxis within a competing gradient of chemoattractants. Anal Chem.

[91] Kingery JR, Hamid T, Lewis RK, Ismahil MA, Bansal SS, Rokosh G, Townes TM, Ildstad ST, Jones SP, Prabhu SD (2017). Leukocyte iNOS is required for inflammation and pathological remodeling in ischemic heart failure. Basic Res Cardiol.

[92] Kohlhaas S, Garden OA, Scudamore C, Turner M, Okkenhaug K, Vigorito E (2009). Cutting edge: the Foxp3 target miR-155 contributes to the development of regulatory T cells. J Immunol.

[93] Kolaczkowska E, Kubes P (2013). Neutrophil recruitment and function in health and inflammation. Nat Rev Immunol.

[94] Kong KY, Owens KS, Rogers JH, Mullenix J, Velu CS, Grimes HL, Dahl R (2010). MIR-23A microRNA cluster inhibits B-cell development. Exp Hematol.

[95] Koretzky GA (2010). Multiple roles of CD4 and CD8 in T cell activation. J Immunol.

[96] Kotzin JJ, Spencer SP, McCright SJ, Kumar DBU, Collet MA, Mowel WK, Elliott EN, Uyar A, Makiya MA, Dunagin MC, Harman CCD, Virtue AT, Zhu S, Bailis W, Stein J, Hughes C, Raj A, Wherry EJ, Goff LA, Klion AD, Rinn JL, Williams A, Flavell RA, Henao-Mejia J (2016). The long non-coding RNA Morrbid regulates Bim and short-lived myeloid cell lifespan. Nature.

[97] Kretzschmar D, Betge S, Windisch A, Pistulli R, Rohm I, Fritzenwanger M, Jung C, Schubert K, Theis B, Petersen I, Drobnik S, Mall G, Figulla HR, Yilmaz A (2012). Recruitment of circulating dendritic cell precursors into the infarcted myocardium and pro-inflammatory response in acute myocardial infarction. Clin Sci (Lond).

[98] Krutzfeldt J, Rajewsky N, Braich R, Rajeev KG, Tuschl T, Manoharan M, Stoffel M (2005). Silencing of microRNAs in vivo with ‘antagomirs’. Nature.

[99] Lambert JM, Lopez EF, Lindsey ML (2008). Macrophage roles following myocardial infarction. Int J Cardiol.

[100] Langston PK, Shibata M, Horng T (2017). Metabolism supports macrophage activation. Front Immunol.

[101] Lanzavecchia A, Sallusto F (2001). The instructive role of dendritic cells on T cell responses: lineages, plasticity and kinetics. Curr Opin Immunol.

[102] Latet SC, Hoymans VY, Van Herck PL, Vrints CJ (2015). The cellular immune system in the post-myocardial infarction repair process. Int J Cardiol.

[103] Laxmanan S, Robertson SW, Wang E, Lau JS, Briscoe DM, Mukhopadhyay D (2005). Vascular endothelial growth factor impairs the functional ability of dendritic cells through Id pathways. Biochem Biophys Res Commun.

[104] Leblond AL, Klinkert K, Martin K, Turner EC, Kumar AH, Browne T, Caplice NM (2015). Systemic and cardiac depletion of M2 macrophage through CSF-1R signaling inhibition alters cardiac function post myocardial infarction. PLoS One.

[105] Legrand D (2016). Overview of lactoferrin as a natural immune modulator. J Pediatr.

[106] Li J, Cai SX, He Q, Zhang H, Friedberg D, Wang F, Redington AN (2018). Intravenous miR-144 reduces left ventricular remodeling after myocardial infarction. Basic Res Cardiol.

[107] Li L, Cong Y, Gao X, Wang Y, Lin P (2017). Differential expression profiles of long non-coding RNAs as potential biomarkers for the early diagnosis of acute myocardial infarction. Oncotarget.

[108] Lin Z, Murtaza I, Wang K, Jiao J, Gao J, Li PF (2009). miR-23a functions downstream of NFATc3 to regulate cardiac hypertrophy. Proc Natl Acad Sci USA.

[109] Liu H, Gao W, Yuan J, Wu C, Yao K, Zhang L, Ma L, Zhu J, Zou Y, Ge J (2016). Exosomes derived from dendritic cells improve cardiac function via activation of CD4(+) T lymphocytes after myocardial infarction. J Mol Cell Cardiol.

[110] Liu J, van Mil A, Vrijsen K, Zhao J, Gao L, Metz CH, Goumans MJ, Doevendans PA, Sluijter JP (2011). MicroRNA-155 prevents necrotic cell death in human cardiomyocyte progenitor cells via targeting RIP1. J Cell Mol Med.

[111] Liu J, Wang H, Li J (2016). Inflammation and inflammatory cells in myocardial infarction and reperfusion injury: a double-edged sword. Clin Med Insights Cardiol.

[112] Liu Z, Yang D, Xie P, Ren G, Sun G, Zeng X, Sun X (2012). MiR-106b and MiR-15b modulate apoptosis and angiogenesis in myocardial infarction. Cell Physiol Biochem.

[113] Liu Z, Ye P, Wang S, Wu J, Sun Y, Zhang A, Ren L, Cheng C, Huang X, Wang K, Deng P, Wu C, Yue Z, Xia J (2015). MicroRNA-150 protects the heart from injury by inhibiting monocyte accumulation in a mouse model of acute myocardial infarction. Circ Cardiovasc Genet.

[114] Long G, Wang F, Duan Q, Chen F, Yang S, Gong W, Wang Y, Chen C, Wang DW (2012). Human circulating microRNA-1 and microRNA-126 as potential novel indicators for acute myocardial infarction. Int J Biol Sci.

[115] Lutz MB, Schuler G (2002). Immature, semi-mature and fully mature dendritic cells: which signals induce tolerance or immunity?. Trends Immunol.

[116] Ma Y, Mouton AJ, Lindsey ML (2018). Cardiac macrophage biology in the steady-state heart, the aging heart, and following myocardial infarction. Transl Res.

[117] Ma Y, Yabluchanskiy A, Iyer RP, Cannon PL, Flynn ER, Jung M, Henry J, Cates CA, Deleon-Pennell KY, Lindsey ML (2016). Temporal neutrophil polarization following myocardial infarction. Cardiovasc Res.

[118] Ma Y, Yabluchanskiy A, Lindsey ML (2013). Neutrophil roles in left ventricular remodeling following myocardial infarction. Fibrogenesis Tissue Repair.

[119] Mannie MD, Curtis AD (2013). Tolerogenic vaccines for multiple sclerosis. Hum Vaccin Immunother.

[120] Martinez-Martinez E, Buonafine M, Boukhalfa I, Ibarrola J, Fernandez-Celis A, Kolkhof P, Rossignol P, Girerd N, Mulder P, Lopez-Andres N, Ouvrard-Pascaud A, Jaisser F (2017). Aldosterone target NGAL (neutrophil gelatinase-associated lipocalin) is involved in cardiac remodeling after myocardial infarction through NFkappaB pathway. Hypertension.

[121] Mathes D, Weirather J, Nordbeck P, Arias-Loza AP, Burkard M, Pachel C, Kerkau T, Beyersdorf N, Frantz S, Hofmann U (2016). CD4(+) Foxp3(+) T-cells contribute to myocardial ischemia-reperfusion injury. J Mol Cell Cardiol.

[122] Matkovich SJ, Edwards JR, Grossenheider TC, de Guzman Strong C, Dorn GW (2014). Epigenetic coordination of embryonic heart transcription by dynamically regulated long noncoding RNAs. Proc Natl Acad Sci USA.

[123] Matsumoto S, Sakata Y, Nakatani D, Suna S, Mizuno H, Shimizu M, Usami M, Sasaki T, Sato H, Kawahara Y, Hamasaki T, Nanto S, Hori M, Komuro I (2012). A subset of circulating microRNAs are predictive for cardiac death after discharge for acute myocardial infarction. Biochem Biophys Res Commun.

[124] Menon V, Lessard D, Yarzebski J, Furman MI, Gore JM, Goldberg RJ (2003). Leukocytosis and adverse hospital outcomes after acute myocardial infarction. Am J Cardiol.

[125] Mocatta TJ, Pilbrow AP, Cameron VA, Senthilmohan R, Frampton CM, Richards AM, Winterbourn CC (2007). Plasma concentrations of myeloperoxidase predict mortality after myocardial infarction. J Am Coll Cardiol.

[126] Molawi K, Wolf Y, Kandalla PK, Favret J, Hagemeyer N, Frenzel K, Pinto AR, Klapproth K, Henri S, Malissen B, Rodewald HR, Rosenthal NA, Bajenoff M, Prinz M, Jung S, Sieweke MH (2014). Progressive replacement of embryo-derived cardiac macrophages with age. J Exp Med.

[127] Montecucco F, Bauer I, Braunersreuther V, Bruzzone S, Akhmedov A, Luscher TF, Speer T, Poggi A, Mannino E, Pelli G, Galan K, Bertolotto M, Lenglet S, Garuti A, Montessuit C, Lerch R, Pellieux C, Vuilleumier N, Dallegri F, Mage J, Sebastian C, Mostoslavsky R, Gayet-Ageron A, Patrone F, Mach F, Nencioni A (2013). Inhibition of nicotinamide phosphoribosyltransferase reduces neutrophil-mediated injury in myocardial infarction. Antioxid Redox Signal.

[128] Morelli AE, Thomson AW (2007). Tolerogenic dendritic cells and the quest for transplant tolerance. Nat Rev Immunol.

[129] Mouton AJ, DeLeon-Pennell KY, Rivera Gonzalez OJ, Flynn ER, Freeman TC, Saucerman JJ, Garrett MR, Ma Y, Harmancey R, Lindsey ML (2018). Mapping macrophage polarization over the myocardial infarction time continuum. Basic Res Cardiol.

[130] Nagai T, Honda S, Sugano Y, Matsuyama TA, Ohta-Ogo K, Asaumi Y, Ikeda Y, Kusano K, Ishihara M, Yasuda S, Ogawa H, Ishibashi-Ueda H, Anzai T (2014). Decreased myocardial dendritic cells is associated with impaired reparative fibrosis and development of cardiac rupture after myocardial infarction in humans. J Am Heart Assoc.

[131] Nahrendorf M, Sosnovik D, Chen JW, Panizzi P, Figueiredo JL, Aikawa E, Libby P, Swirski FK, Weissleder R (2008). Activatable magnetic resonance imaging agent reports myeloperoxidase activity in healing infarcts and noninvasively detects the antiinflammatory effects of atorvastatin on ischemia-reperfusion injury. Circulation.

[132] Nahrendorf M, Swirski FK (2016). Innate immune cells in ischaemic heart disease: does myocardial infarction beget myocardial infarction?. Eur Heart J.

[133] Nahrendorf M, Swirski FK, Aikawa E, Stangenberg L, Wurdinger T, Figueiredo JL, Libby P, Weissleder R, Pittet MJ (2007). The healing myocardium sequentially mobilizes two monocyte subsets with divergent and complementary functions. J Exp Med.

[134] Nalbant A, Cinemre H, Kaya T, Varim C, Varim P, Tamer A (2016). Neutrophil to lymphocyte ratio might help prediction of acute myocardial infarction in patients with elevated serum creatinine. Pak J Med Sci.

[135] Nazari-Jahantigh M, Wei Y, Noels H, Akhtar S, Zhou Z, Koenen RR, Heyll K, Gremse F, Kiessling F, Grommes J, Weber C, Schober A (2012). MicroRNA-155 promotes atherosclerosis by repressing Bcl6 in macrophages. J Clin Invest.

[136] Ndrepepa G, Kastrati A (2013). Intravenous beta-blockers in primary percutaneous coronary intervention: new hope for an old therapy. Circulation.

[137] Nunez J, Nunez E, Bodi V, Sanchis J, Minana G, Mainar L, Santas E, Merlos P, Rumiz E, Darmofal H, Heatta AM, Llacer A (2008). Usefulness of the neutrophil to lymphocyte ratio in predicting long-term mortality in ST segment elevation myocardial infarction. Am J Cardiol.

[138] O’Donoghue M, Morrow DA, Cannon CP, Guo W, Murphy SA, Gibson CM, Sabatine MS (2008). Association between baseline neutrophil count, clopidogrel therapy, and clinical and angiographic outcomes in patients with ST-elevation myocardial infarction receiving fibrinolytic therapy. Eur Heart J.

[139] Oh YB, Ahn M, Lee SM, Koh HW, Lee SH, Kim SH, Park BH (2013). Inhibition of Janus activated kinase-3 protects against myocardial ischemia and reperfusion injury in mice. Exp Mol Med.

[140] Okubo K, Kamiya M, Urano Y, Nishi H, Herter JM, Mayadas T, Hirohama D, Suzuki K, Kawakami H, Tanaka M, Kurosawa M, Kagaya S, Hishikawa K, Nangaku M, Fujita T, Hayashi M, Hirahashi J (2016). Lactoferrin suppresses neutrophil extracellular traps release in inflammation. EBioMedicine.

[141] Ong SB, Hernandez-Resendiz S, Crespo-Avilan GE, Mukhametshina RT, Kwek XY, Cabrera-Fuentes HA, Hausenloy DJ (2018). Inflammation following acute myocardial infarction: multiple players, dynamic roles, and novel therapeutic opportunities. Pharmacol Ther.

[142] Ortega-Gomez A, Perretti M, Soehnlein O (2013). Resolution of inflammation: an integrated view. EMBO Mol Med.

[143] Ouimet M, Ediriweera HN, Gundra UM, Sheedy FJ, Ramkhelawon B, Hutchison SB, Rinehold K, van Solingen C, Fullerton MD, Cecchini K, Rayner KJ, Steinberg GR, Zamore PD, Fisher EA, Loke P, Moore KJ (2015). MicroRNA-33-dependent regulation of macrophage metabolism directs immune cell polarization in atherosclerosis. J Clin Invest.

[144] Oyama J, Blais C, Liu X, Pu M, Kobzik L, Kelly RA, Bourcier T (2004). Reduced myocardial ischemia-reperfusion injury in toll-like receptor 4-deficient mice. Circulation.

[145] Palevski D, Levin-Kotler LP, Kain D, Naftali-Shani N, Landa N, Ben-Mordechai T, Konfino T, Holbova R, Molotski N, Rosin-Arbesfeld R, Lang RA, Leor J (2017). Loss of macrophage Wnt secretion improves remodeling and function after myocardial infarction in mice. J Am Heart Assoc.

[146] Pillay J, den Braber I, Vrisekoop N, Kwast LM, de Boer RJ, Borghans JA, Tesselaar K, Koenderman L (2010). In vivo labeling with 2H_2_O reveals a human neutrophil lifespan of 5.4 days. Blood.

[147] Pillay J, Kamp VM, van Hoffen E, Visser T, Tak T, Lammers JW, Ulfman LH, Leenen LP, Pickkers P, Koenderman L (2012). A subset of neutrophils in human systemic inflammation inhibits T cell responses through Mac-1. J Clin Invest.

[148] Pinto AR, Paolicelli R, Salimova E, Gospocic J, Slonimsky E, Bilbao-Cortes D, Godwin JW, Rosenthal NA (2012). An abundant tissue macrophage population in the adult murine heart with a distinct alternatively-activated macrophage profile. PLoS One.

[149] Porrello ER, Olson EN (2014). A neonatal blueprint for cardiac regeneration. Stem Cell Res.

[150] Randolph GJ, Ochando J, Partida-Sanchez S (2008). Migration of dendritic cell subsets and their precursors. Annu Rev Immunol.

[151] Rao DS, O’Connell RM, Chaudhuri AA, Garcia-Flores Y, Geiger TL, Baltimore D (2010). MicroRNA-34a perturbs B lymphocyte development by repressing the forkhead box transcription factor Foxp1. Immunity.

[152] Reddy MA, Chen Z, Park JT, Wang M, Lanting L, Zhang Q, Bhatt K, Leung A, Wu X, Putta S, Saetrom P, Devaraj S, Natarajan R (2014). Regulation of inflammatory phenotype in macrophages by a diabetes-induced long noncoding RNA. Diabetes.

[153] Rienks M, Carai P, Bitsch N, Schellings M, Vanhaverbeke M, Verjans J, Cuijpers I, Heymans S, Papageorgiou A (2017). Sema3A promotes the resolution of cardiac inflammation after myocardial infarction. Basic Res Cardiol.

[154] Roberts TC, Wood MJ (2013). Therapeutic targeting of non-coding RNAs. Essays Biochem.

[155] Rogacev KS, Cremers B, Zawada AM, Seiler S, Binder N, Ege P, Grosse-Dunker G, Heisel I, Hornof F, Jeken J, Rebling NM, Ulrich C, Scheller B, Bohm M, Fliser D, Heine GH (2012). CD14^++^CD16^+^ monocytes independently predict cardiovascular events: a cohort study of 951 patients referred for elective coronary angiography. J Am Coll Cardiol.

[156] Sallusto F, Palermo B, Lenig D, Miettinen M, Matikainen S, Julkunen I, Forster R, Burgstahler R, Lipp M, Lanzavecchia A (1999). Distinct patterns and kinetics of chemokine production regulate dendritic cell function. Eur J Immunol.

[157] Schloss MJ, Horckmans M, Nitz K, Duchene J, Drechsler M, Bidzhekov K, Scheiermann C, Weber C, Soehnlein O, Steffens S (2016). The time-of-day of myocardial infarction onset affects healing through oscillations in cardiac neutrophil recruitment. EMBO Mol Med.

[158] Schwarz BA, Bhandoola A (2006). Trafficking from the bone marrow to the thymus: a prerequisite for thymopoiesis. Immunol Rev.

[159] Shantsila E, Wrigley B, Tapp L, Apostolakis S, Montoro-Garcia S, Drayson MT, Lip GY (2011). Immunophenotypic characterization of human monocyte subsets: possible implications for cardiovascular disease pathophysiology. J Thromb Haemost.

[160] Sharir R, Semo J, Shimoni S, Ben-Mordechai T, Landa-Rouben N, Maysel-Auslender S, Shaish A, Entin-Meer M, Keren G, George J (2014). Experimental myocardial infarction induces altered regulatory T cell hemostasis, and adoptive transfer attenuates subsequent remodeling. PLoS One.

[161] Shimizu T, Suzuki S, Sato A, Nakamura Y, Ikeda K, Saitoh S, Misaka S, Shishido T, Kubota I, Takeishi Y (2015). Cardio-protective effects of pentraxin 3 produced from bone marrow-derived cells against ischemia/reperfusion injury. J Mol Cell Cardiol.

[162] Shiraishi M, Shintani Y, Shintani Y, Ishida H, Saba R, Yamaguchi A, Adachi H, Yashiro K, Suzuki K (2016). Alternatively activated macrophages determine repair of the infarcted adult murine heart. J Clin Invest.

[163] Sieweke MH, Allen JE (2013). Beyond stem cells: self-renewal of differentiated macrophages. Science.

[164] Soehnlein O (2012). Multiple roles for neutrophils in atherosclerosis. Circ Res.

[165] Soehnlein O, Lindbom L (2010). Phagocyte partnership during the onset and resolution of inflammation. Nat Rev Immunol.

[166] Steinman RM, Inaba K (1999). Myeloid dendritic cells. J Leukoc Biol.

[167] Stoop JN, Robinson JH, Hilkens CM (2011). Developing tolerogenic dendritic cell therapy for rheumatoid arthritis: what can we learn from mouse models?. Ann Rheum Dis.

[168] Su SA, Ma H, Shen L, Xiang MX, Wang JA (2013). Interleukin-17 and acute coronary syndrome. J Zhejiang Univ Sci B.

[169] Sugimoto MA, Vago JP, Teixeira MM, Sousa LP (2016). Annexin A1 and the resolution of inflammation: modulation of neutrophil recruitment, apoptosis, and clearance. J Immunol Res.

[170] Summers deLuca L, Gommerman JL (2012). Fine-tuning of dendritic cell biology by the TNF superfamily. Nat Rev Immunol.

[171] Swirski FK, Nahrendorf M, Etzrodt M, Wildgruber M, Cortez-Retamozo V, Panizzi P, Figueiredo JL, Kohler RH, Chudnovskiy A, Waterman P, Aikawa E, Mempel TR, Libby P, Weissleder R, Pittet MJ (2009). Identification of splenic reservoir monocytes and their deployment to inflammatory sites. Science.

[172] Swirski FK, Robbins CS, Nahrendorf M (2016). Development and function of arterial and cardiac macrophages. Trends Immunol.

[173] Syrjala H, Surcel HM, Ilonen J (1991). Low CD4/CD8 T lymphocyte ratio in acute myocardial infarction. Clin Exp Immunol.

[174] Tae YuH, Youn JC, Lee J, Park S, Chi HS, Lee J, Choi C, Park S, Choi D, Ha JW, Shin EC (2015). Characterization of CD8(+)CD57(+) T cells in patients with acute myocardial infarction. Cell Mol Immunol.

[175] Taibi F, Metzinger-Le Meuth V, Massy ZA, Metzinger L (2014). miR-223: an inflammatory oncomiR enters the cardiovascular field. Biochim Biophys Acta.

[176] Takahashi M (2014). NLRP3 inflammasome as a novel player in myocardial infarction. Int Heart J.

[177] Teng Y, Zhang R, Liu C, Zhou L, Wang H, Zhuang W, Huang Y, Hong Z (2015). miR-143 inhibits interleukin-13-induced inflammatory cytokine and mucus production in nasal epithelial cells from allergic rhinitis patients by targeting IL13Ralpha1. Biochem Biophys Res Commun.

[178] Thomson AW (2010). Tolerogenic dendritic cells: all present and correct?. Am J Transplant.

[179] Thum T (2012). MicroRNA therapeutics in cardiovascular medicine. EMBO Mol Med.

[180] Tian X, Tian J, Tang X, Ma J, Wang S (2016). Long non-coding RNAs in the regulation of myeloid cells. J Hematol Oncol.

[181] Timmers L, Sluijter JP, van Keulen JK, Hoefer IE, Nederhoff MG, Goumans MJ, Doevendans PA, van Echteld CJ, Joles JA, Quax PH, Piek JJ, Pasterkamp G, de Kleijn DP (2008). Toll-like receptor 4 mediates maladaptive left ventricular remodeling and impairs cardiac function after myocardial infarction. Circ Res.

[182] Tomczyk M, Kraszewska I, Szade K, Bukowska-Strakova K, Meloni M, Jozkowicz A, Dulak J, Jazwa A (2017). Splenic Ly6C(hi) monocytes contribute to adverse late post-ischemic left ventricular remodeling in heme oxygenase-1 deficient mice. Basic Res Cardiol.

[183] Tracchi I, Ghigliotti G, Mura M, Garibaldi S, Spallarossa P, Barisione C, Boasi V, Brunelli M, Corsiglia L, Barsotti A, Brunelli C (2009). Increased neutrophil lifespan in patients with congestive heart failure. Eur J Heart Fail.

[184] Van den Bossche J, Baardman J, de Winther MP (2015). Metabolic characterization of polarized M1 and M2 bone marrow-derived macrophages using real-time extracellular flux analysis. J Vis Exp.

[185] Van den Bossche J, Baardman J, Otto NA, van der Velden S, Neele AE, van den Berg SM, Luque-Martin R, Chen HJ, Boshuizen MC, Ahmed M, Hoeksema MA, de Vos AF, de Winther MP (2016). Mitochondrial dysfunction prevents repolarization of inflammatory macrophages. Cell Rep.

[186] Van der Borght K, Scott CL, Nindl V, Bouche A, Martens L, Sichien D, Van Moorleghem J, Vanheerswynghels M, De Prijck S, Saeys Y, Ludewig B, Gillebert T, Guilliams M, Carmeliet P, Lambrecht BN (2017). Myocardial infarction primes autoreactive T cells through activation of dendritic cells. Cell Rep.

[187] van der Laan AM, Nahrendorf M, Piek JJ (2013). Republished: healing and adverse remodelling after acute myocardial infarction: role of the cellular immune response. Postgrad Med J.

[188] Van Vre EA, Van Brussel I, Bosmans JM, Vrints CJ, Bult H (2011). Dendritic cells in human atherosclerosis: from circulation to atherosclerotic plaques. Mediators Inflamm.

[189] Vasilyev N, Williams T, Brennan ML, Unzek S, Zhou X, Heinecke JW, Spitz DR, Topol EJ, Hazen SL, Penn MS (2005). Myeloperoxidase-generated oxidants modulate left ventricular remodeling but not infarct size after myocardial infarction. Circulation.

[190] Vausort M, Wagner DR, Devaux Y (2014). Long noncoding RNAs in patients with acute myocardial infarction. Circ Res.

[191] Vengen IT, Dale AC, Wiseth R, Midthjell K, Videm V (2010). Lactoferrin is a novel predictor of fatal ischemic heart disease in diabetes mellitus type 2: long-term follow-up of the HUNT 1 study. Atherosclerosis.

[192] Vigneau S, Rohrlich PS, Brahic M, Bureau JF (2003). Tmevpg1, a candidate gene for the control of Theiler’s virus persistence, could be implicated in the regulation of gamma interferon. J Virol.

[193] Vinten-Johansen J (2004). Involvement of neutrophils in the pathogenesis of lethal myocardial reperfusion injury. Cardiovasc Res.

[194] Vorobjeva NV, Pinegin BV (2014). Neutrophil extracellular traps: mechanisms of formation and role in health and disease. Biochemistry (Mosc).

[195] Wakkach A, Fournier N, Brun V, Breittmayer JP, Cottrez F, Groux H (2003). Characterization of dendritic cells that induce tolerance and T regulatory 1 cell differentiation in vivo. Immunity.

[196] Wan E, Yeap XY, Dehn S, Terry R, Novak M, Zhang S, Iwata S, Han X, Homma S, Drosatos K, Lomasney J, Engman DM, Miller SD, Vaughan DE, Morrow JP, Kishore R, Thorp EB (2013). Enhanced efferocytosis of apoptotic cardiomyocytes through myeloid-epithelial-reproductive tyrosine kinase links acute inflammation resolution to cardiac repair after infarction. Circ Res.

[197] Wang C, Zhang C, Liu L, Xi A, Chen B, Li Y, Du J (2017). Macrophage-derived mir-155-containing exosomes suppress fibroblast proliferation and promote fibroblast inflammation during cardiac injury. Mol Ther.

[198] Wang F, Long G, Zhao C, Li H, Chaugai S, Wang Y, Chen C, Wang DW (2014). Atherosclerosis-related circulating miRNAs as novel and sensitive predictors for acute myocardial infarction. PLoS One.

[199] Wang K, Long B, Liu F, Wang JX, Liu CY, Zhao B, Zhou LY, Sun T, Wang M, Yu T, Gong Y, Liu J, Dong YH, Li N, Li PF (2016). A circular RNA protects the heart from pathological hypertrophy and heart failure by targeting miR-223. Eur Heart J.

[200] Wang P, Xue Y, Han Y, Lin L, Wu C, Xu S, Jiang Z, Xu J, Liu Q, Cao X (2014). The STAT3-binding long noncoding RNA lnc-DC controls human dendritic cell differentiation. Science.

[201] Wang X, Ha T, Liu L, Zou J, Zhang X, Kalbfleisch J, Gao X, Williams D, Li C (2013). Increased expression of microRNA-146a decreases myocardial ischaemia/reperfusion injury. Cardiovasc Res.

[202] Wang X, Huang W, Yang Y, Wang Y, Peng T, Chang J, Caldwell CC, Zingarelli B, Fan GC (2014). Loss of duplexmiR-223 (5p and 3p) aggravates myocardial depression and mortality in polymicrobial sepsis. Biochim Biophys Acta.

[203] Wang Z (2011). The concept of multiple-target anti-miRNA antisense oligonucleotide technology. Methods Mol Biol.

[204] Ward JR, Heath PR, Catto JW, Whyte MK, Milo M, Renshaw SA (2011). Regulation of neutrophil senescence by microRNAs. PLoS One.

[205] Wei G, Jie Y, Haibo L, Chaoneng W, Dong H, Jianbing Z, Junjie G, Leilei M, Hongtao S, Yunzeng Z, Junbo G (2017). Dendritic cells derived exosomes migration to spleen and induction of inflammation are regulated by CCR7. Sci Rep.

[206] Weirather J, Hofmann UD, Beyersdorf N, Ramos GC, Vogel B, Frey A, Ertl G, Kerkau T, Frantz S (2014). Foxp3^+^CD4^+^ T cells improve healing after myocardial infarction by modulating monocyte/macrophage differentiation. Circ Res.

[207] Weisheit C, Zhang Y, Faron A, Kopke O, Weisheit G, Steinstrasser A, Frede S, Meyer R, Boehm O, Hoeft A, Kurts C, Baumgarten G (2014). Ly6C(low) and not Ly6C(high) macrophages accumulate first in the heart in a model of murine pressure-overload. PLoS One.

[208] Wynn TA, Vannella KM (2016). Macrophages in tissue repair, regeneration, and fibrosis. Immunity.

[209] Xia H, Qi Y, Ng SS, Chen X, Chen S, Fang M, Li D, Zhao Y, Ge R, Li G, Chen Y, He ML, Kung HF, Lai L, Lin MC (2009). MicroRNA-15b regulates cell cycle progression by targeting cyclins in glioma cells. Biochem Biophys Res Commun.

[210] Xiao C, Srinivasan L, Calado DP, Patterson HC, Zhang B, Wang J, Henderson JM, Kutok JL, Rajewsky K (2008). Lymphoproliferative disease and autoimmunity in mice with increased miR-17-92 expression in lymphocytes. Nat Immunol.

[211] Yan H, Ma F, Zhang Y, Wang C, Qiu D, Zhou K, Hua Y, Li Y (2017). miRNAs as biomarkers for diagnosis of heart failure: a systematic review and meta-analysis. Medicine (Baltimore).

[212] Yan X, Anzai A, Katsumata Y, Matsuhashi T, Ito K, Endo J, Yamamoto T, Takeshima A, Shinmura K, Shen W, Fukuda K, Sano M (2013). Temporal dynamics of cardiac immune cell accumulation following acute myocardial infarction. J Mol Cell Cardiol.

[213] Yan X, Zhang H, Fan Q, Hu J, Tao R, Chen Q, Iwakura Y, Shen W, Lu L, Zhang Q, Zhang R (2017). Dectin-2 deficiency modulates Th1 differentiation and improves wound healing after myocardial infarction. Circ Res.

[214] Yilmaz A, Weber J, Cicha I, Stumpf C, Klein M, Raithel D, Daniel WG, Garlichs CD (2006). Decrease in circulating myeloid dendritic cell precursors in coronary artery disease. J Am Coll Cardiol.

[215] Zangrando J, Zhang L, Vausort M, Maskali F, Marie PY, Wagner DR, Devaux Y (2014). Identification of candidate long non-coding RNAs in response to myocardial infarction. BMC Genom.

[216] Zemmour D, Pratama A, Loughhead SM, Mathis D, Benoist C (2017). Flicr, a long noncoding RNA, modulates Foxp3 expression and autoimmunity. Proc Natl Acad Sci USA.

[217] Zhang DD, Wang WT, Xiong J, Xie XM, Cui SS, Zhao ZG, Li MJ, Zhang ZQ, Hao DL, Zhao X, Li YJ, Wang J, Chen HZ, Lv X, Liu DP (2017). Long noncoding RNA LINC00305 promotes inflammation by activating the AHRR-NF-kappaB pathway in human monocytes. Sci Rep.

[218] Zhang S, Zhang M, Goldstein S, Li Y, Ge J, He B, Ruiz G (2013). The effect of c-fos on acute myocardial infarction and the significance of metoprolol intervention in a rat model. Cell Biochem Biophys.

[219] Zhang Y, Zhang M, Li X, Tang Z, Wang X, Zhong M, Suo Q, Zhang Y, Lv K (2016). Silencing microRNA-155 attenuates cardiac injury and dysfunction in viral myocarditis via promotion of M2 phenotype polarization of macrophages. Sci Rep.

[220] Zhang Y, Zhang M, Zhong M, Suo Q, Lv K (2013). Expression profiles of miRNAs in polarized macrophages. Int J Mol Med.

[221] Zhao W, Zhao SP, Zhao YH (2015). MicroRNA-143/-145 in cardiovascular diseases. Biomed Res Int.

[222] Zhu B, Ye J, Nie Y, Ashraf U, Zohaib A, Duan X, Fu ZF, Song Y, Chen H, Cao S (2015). MicroRNA-15b modulates japanese encephalitis virus-mediated inflammation via targeting RNF125. J Immunol.

[223] Zhu J, Paul WE (2008). CD4 T cells: fates, functions, and faults. Blood.

[224] Zhu J, Yamane H, Paul WE (2010). Differentiation of effector CD4 T cell populations (*). Annu Rev Immunol.

[225] Zhu J, Yao K, Guo J, Shi H, Ma L, Wang Q, Liu H, Gao W, Sun A, Zou Y, Ge J (2017). miR-181a and miR-150 regulate dendritic cell immune inflammatory responses and cardiomyocyte apoptosis via targeting JAK1-STAT1/c-Fos pathway. J Cell Mol Med.

[226] Zhu R, Sun H, Yu K, Zhong Y, Shi H, Wei Y, Su X, Xu W, Luo Q, Zhang F, Zhu Z, Meng K, Zhao X, Liu Y, Mao Y, Cheng P, Mao X, Zeng Q (2016). Interleukin-37 and dendritic cells treated with Interleukin-37 plus troponin I ameliorate cardiac remodeling after myocardial infarction. J Am Heart Assoc.

[227] Zidar N, Jeruc J, Balazic J, Stajer D (2005). Neutrophils in human myocardial infarction with rupture of the free wall. Cardiovasc Pathol.

[228] Ziegler-Heitbrock L, Ancuta P, Crowe S, Dalod M, Grau V, Hart DN, Leenen PJ, Liu YJ, MacPherson G, Randolph GJ, Scherberich J, Schmitz J, Shortman K, Sozzani S, Strobl H, Zembala M, Austyn JM, Lutz MB (2010). Nomenclature of monocytes and dendritic cells in blood. Blood.

[229] Zlatanova I, Pinto C, Silvestre JS (2016). Immune modulation of cardiac repair and regeneration: the art of mending broken hearts. Front Cardiovasc Med.

[230] Zouggari Y, Ait-Oufella H, Bonnin P, Simon T, Sage AP, Guerin C, Vilar J, Caligiuri G, Tsiantoulas D, Laurans L, Dumeau E, Kotti S, Bruneval P, Charo IF, Binder CJ, Danchin N, Tedgui A, Tedder TF, Silvestre JS, Mallat Z (2013). B lymphocytes trigger monocyte mobilization and impair heart function after acute myocardial infarction. Nat Med.

